# A novel single-cell model reveals ferroptosis-associated biomarkers for individualized therapy and prognostic prediction in hepatocellular carcinoma

**DOI:** 10.1186/s12915-024-01931-z

**Published:** 2024-06-10

**Authors:** Qiong Zhou, Chunyu Tao, Yuli Ge, Jiakai Yuan, Fan Pan, Xinrong Lin, Rui Wang

**Affiliations:** 1 Department of Medical Oncology, Nanjing Jinling Hospital, Affiliated Hospital of Medical School, Nanjing University, Nanjing, Jiangsu Province 210093 PR China; 2 Department of Medical Oncology, Jinling Clinical Medical College, Nanjing University of Chinese Medicine, Nanjing, Jiangsu Province 210023 PR China

**Keywords:** Hepatocellular carcinoma, Ferroptosis, Single-cell sequencing, Immune response, Biomarkers, Predictive model

## Abstract

**Background:**

Hepatocellular carcinoma (HCC) is a prevalent malignancy with a pressing need for improved therapeutic response and prognosis prediction. This study delves into a novel predictive model related to ferroptosis, a regulated cell death mechanism disrupting metabolic processes.

**Results:**

Single-cell sequencing data analysis identified subpopulations of HCC cells exhibiting activated ferroptosis and distinct gene expression patterns compared to normal tissues. Utilizing the LASSO-Cox algorithm, we constructed a model with 10 single-cell biomarkers associated with ferroptosis, namely STMN1, S100A10, FABP5, CAPG, RGCC, ENO1, ANXA5, UTRN, CXCR3, and ITM2A. Comprehensive analyses using these biomarkers revealed variations in immune infiltration, tumor mutation burden, drug sensitivity, and biological functional profiles between risk groups. Specific associations were established between particular immune cell subtypes and certain gene expression patterns. Treatment response analyses indicated potential benefits from anti-tumor immune therapy for the low-risk group and chemotherapy advantages for the high-risk group.

**Conclusions:**

The integration of this single-cell level model with clinicopathological features enabled accurate overall survival prediction and effective risk stratification in HCC patients. Our findings illuminate the potential of ferroptosis-related genes in tailoring therapy and prognosis prediction for HCC, offering novel insights into the intricate interplay among ferroptosis, immune response, and HCC progression.

**Supplementary Information:**

The online version contains supplementary material available at 10.1186/s12915-024-01931-z.

## Background

Primary liver cancer ranks among the most prevalent malignant neoplasms on a global scale, representing a significant menace to human health owing to its high incidence and mortality rates [1]. Hepatocellular carcinoma (HCC) represents its predominant biological subtype, with 50% of HCC cases originating from China [2]. Despite the potential life-saving benefits of curative surgery, radiotherapy, transcatheter arterial chemoembolization, and targeted therapy in the management of HCC, a considerable proportion of patients unfortunately receive a diagnosis when they are already in advanced stages or exhibit insensitivity or resistance to drug treatment [3, 4]. Despite efforts, the overall prognosis for HCC is still unsatisfactory, with a 5-year survival rate of less than 20% [5]. As of now, there is still a lack of widely established predictive models that can reliably forecast patients’ survival prognosis and assist clinical practitioners in making treatment decisions, surpassing the traditional TNM staging system and Child–Pugh scoring system based on liver function assessment [6–8]. Therefore, a comprehensive understanding of the molecular biology mechanisms driving HCC is essential for exploring innovative therapeutic strategies and identifying prognostic biomarkers that can accurately predict survival outcomes.

Ferroptosis is a programmed cell death mechanism closely associated with disruptions in intracellular iron metabolism, lipid metabolism, and redox homeostasis [9, 10]. Multiple studies have indicated the significant regulatory role of ferroptosis in HCC, including its ability to inhibit tumor cell proliferation and modulate the tumor immune microenvironment (TIME) [11, 12]. The recent progress in single-cell transcriptomic technology has significantly mitigated the limitations associated with bulk RNA sequencing, specifically in capturing the diversity among cells [13]. This advanced technique offers a higher level of precision in identifying distinct cell types and their states, thus serving as a formidable tool for investigating potential mechanisms involved in ferroptosis and the pathogenesis of HCC.

In this study, we conducted an integrative analysis of RNA sequencing data from HCC single-cell datasets obtained from the Gene Expression Omnibus (GEO). Additionally, bulk transcriptome data containing clinical and prognostic information were sourced from The Cancer Genome Atlas (TCGA). By utilizing bioinformatics techniques, we revealed the susceptibility of populations with HCC to therapy and identified prognostic biomarkers that can aid in predicting survival outcomes. Furthermore, we established a clinical predictive model linked to ferroptosis and investigated the influence of tumor mutation burden (TMB) and TIME in individuals with HCC who possess different risk profiles. It is worth highlighting that with the aid of single-cell analysis, our research has shed light on the biological mechanisms of ferroptosis and provided insights into precision medicine strategies in HCC.

## Results

### Profiling the GSE140228 cohort through scRNA-seq

**Fig. 1:**
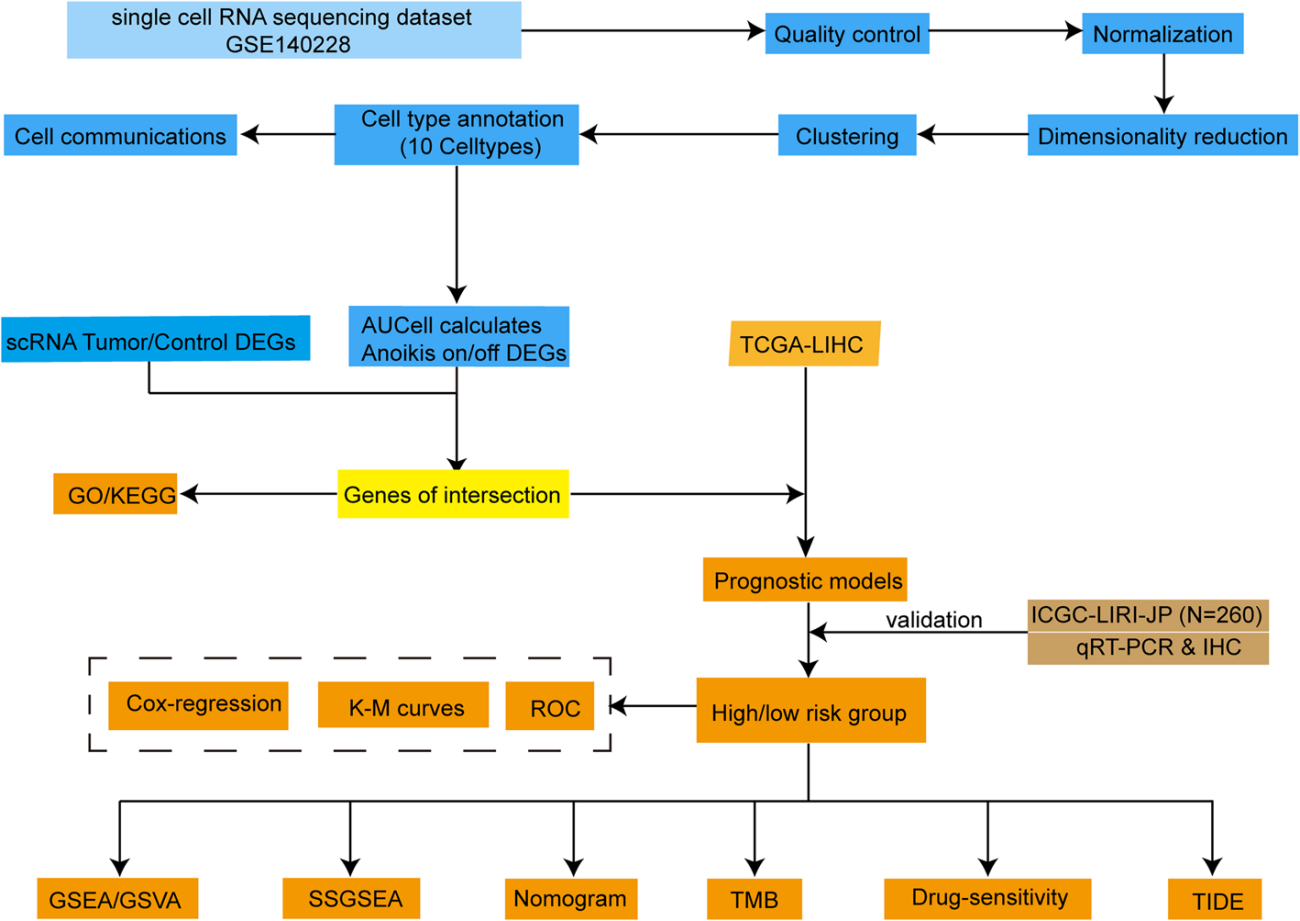
Workflow of this study

**Fig. 2:**
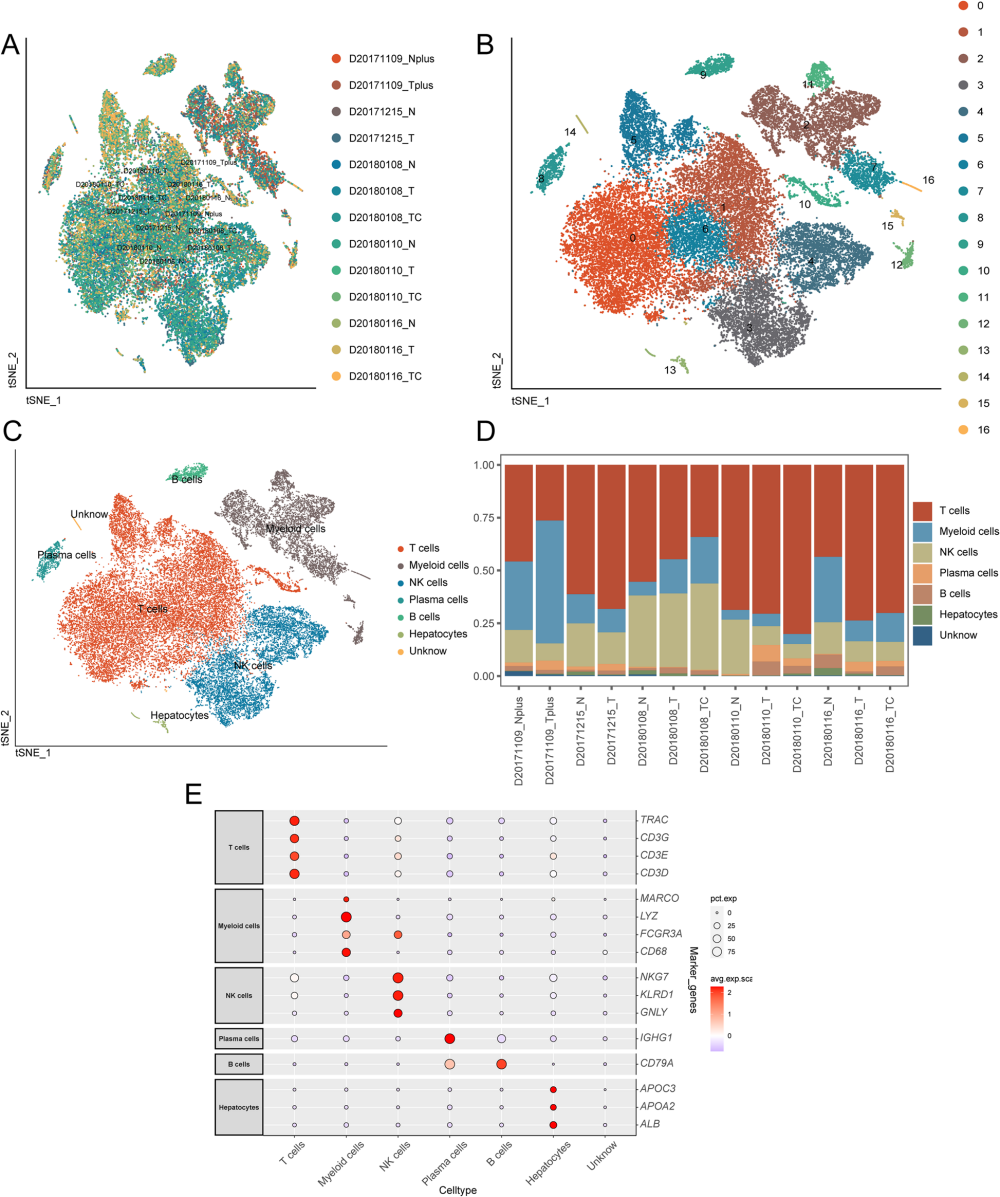
Subsets within cell types identified through single-cell RNA sequencing. **A** Distribution of HCC and normal control samples as shown in the t-SNE plot. **B** Distribution of clusters of HCC cells depicted in the t-SNE plot. **C** Annotation results of HCC cell subgroups indicated by the t-SNE plot. **D** Distribution of cell types between HCC and normal control groups displayed in the cumulative histogram. **E** Expression of marker genes in each cell type

Revealed in Fig. 1 is a comprehensive portrayal of the cohort and study workflow, providing a holistic understanding of the research process. Following preliminary quality control assessment and twin cell removal, a total of 31,869 cells were obtained from the single-cell HCC dataset GSE140228. As illustrated in Fig. 2A, this study encompassed a combined total of 13 samples comprising cancer and control groups. The cellular distribution between groups exhibited relative uniformity, indicating the absence of noticeable batch effects among the samples. Based on the gene expression traits of each cluster, we classified all cells into 17 discrete groups (Fig. 2B). Different cell types were then annotated using cell-specific biomarkers. As depicted in Fig. 2C, six cell types were identified: T cells, myeloid cells, NK cells, plasma cells, B cells, and hepatocytes. However, cluster 14 could not be distinguished and was denoted as “unknown.” The proportions of each distinct cell type within the groups are displayed in Fig. 2D. Specific genes associated with each cell type were visualized through dot plots (Fig. 2E).

### Identification and developmental trajectory analysis of ferroptosis-active cell subgroups

**Fig. 3:**
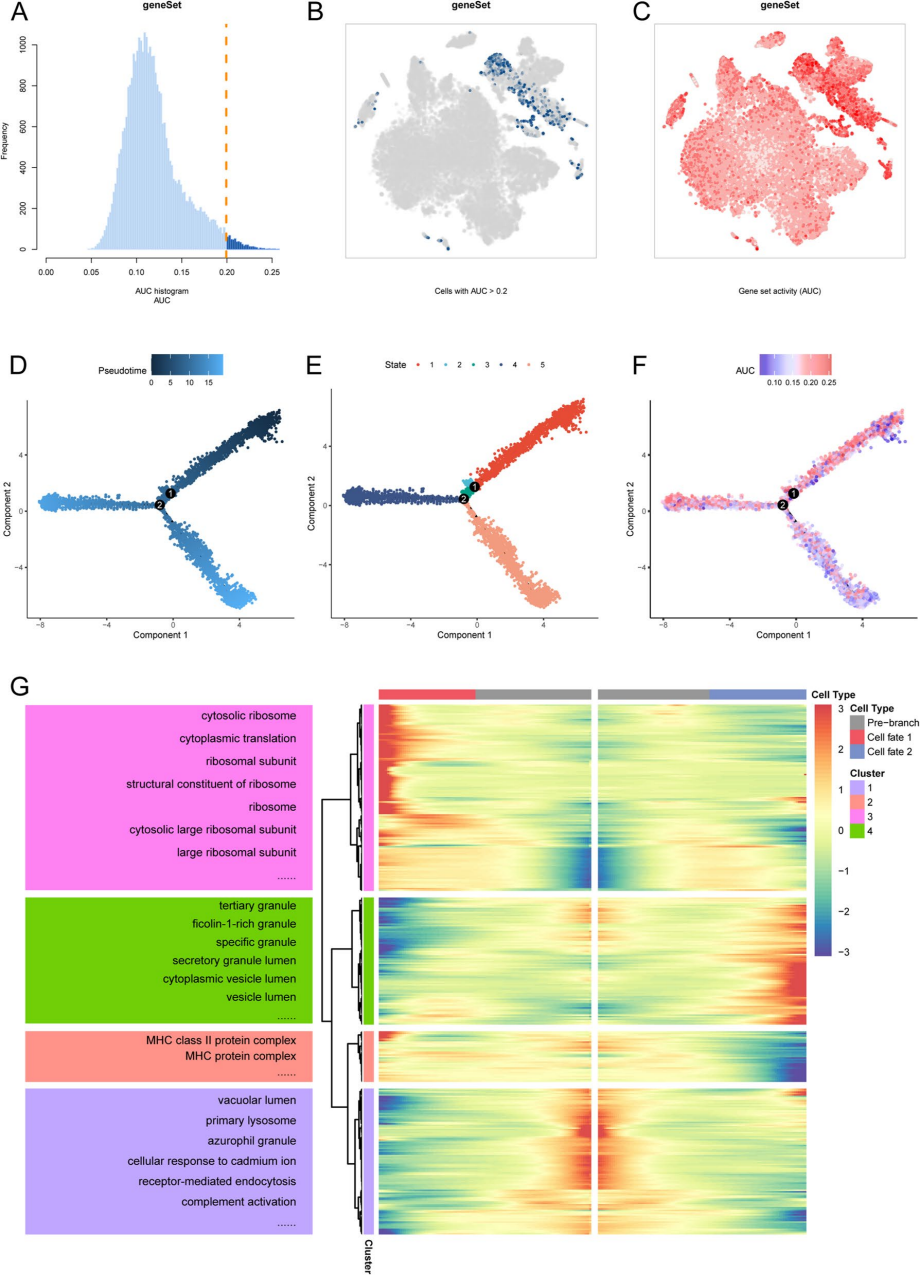
Identification and developmental trajectory analysis of ferroptosis-activated myeloid cells. **A** AUC scores of ferroptosis-related genes with a threshold of 0.2. **B** tSNE plot displays the distribution of cells with AUC values greater than the threshold. **C** tSNE color plot represents the activity scores of cells. Brighter colors indicate higher activity. **D** Pseudotime color gradient transitions from dark blue to light blue. **E** Pseudo-time trajectory is divided into five distinct states by Monocle2. **F** Pseudo-time trajectory based on AUCell scores, with darker colors indicating higher ferroptosis activity. **G** Heatmap reveals differentially expressed genes (DEGs) in different branches (cell fate). Enriched GO pathways for different gene clusters are displayed on the left

To identify active cell subsets demonstrating ferroptosis activity at the single-cell level, we employed an optimal threshold determined by the expression levels of ferroptosis-related genes (Additional file 1: Table S1) across various cell populations within the investigated cohort. Cells surpassing this threshold were considered actively engaged in ferroptosis. The resultant analysis revealed 585 cells displaying ferroptosis activity, as depicted in Fig. 3A. Cell populations with an AUC value greater than 0.2 were categorized as highly active in ferroptosis (Additional file 2: Table S2), while those with a value lower than 0.2 were classified as having low ferroptosis activity. The t-SNE plots presented in Fig. 3B, C visualize the distribution of active cells, highlighting myeloid cells as the most active subset.

For the myeloid cells cluster, we established a pseudotime cell trajectory (Fig. 3D) to investigate the dynamics and gene expression programs underlying ferroptosis. In fact, the transitional states within the trajectory reveal distinct processes (Fig. 3E). Profound variations in AUCell scores are evident throughout the trajectory, wherein Cellfate1 (state 4) demonstrates a sustained level of ferroptosis activity that remains relatively stable compared to the pre-branch phase. Conversely, Cellfate2 (state 5) exhibits a significant reduction in ferroptosis activity, as illustrated in Fig. 3F. The differential gene expression analysis conducted on cells before and after the branching point (Additional file 3: Figure S1, Additional file 4: Figure S2) provides valuable insights into the biological relevance of ferroptosis-related characteristics in myeloid cells. To elucidate the molecular basis of these transitions, we explored the genes that govern the branching of cell fate in ferroptosis. Genes highly expressed in the pre-branch were primarily enriched in the “vacuolar lumen,” “receptor-mediated endocytosis,” and “blood microparticle” GO biological process pathways. Genes enriched in pathways such as “tertiary granule,” “positive regulation of response to external stimulus,” “specific granule,” and “ficolin-1-rich granule” were highly expressed in cell branch 2, while genes involved in pathways like “MHC class II protein complex,” “cytosolic ribosome,” and “cytoplasmic translation” were highly expressed in cell branch 1 (Fig. 3G) (Additional file 5: Table S3).

### Intercellular communication between specific immune subpopulations with myeloid cells involved in ferroptosis

**Fig. 4:**
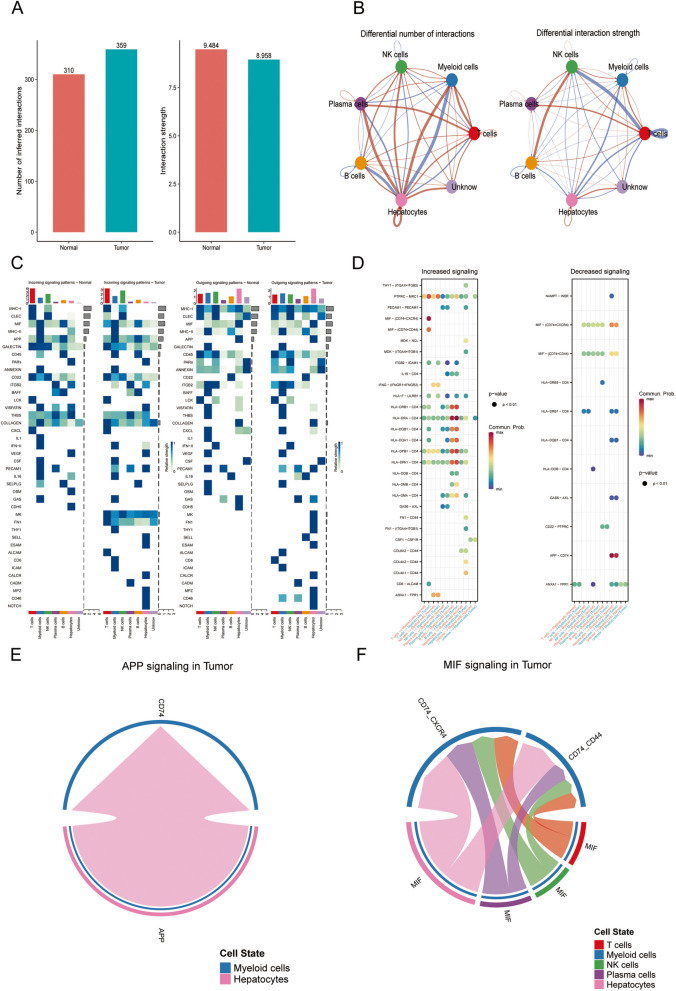
Cell–cell communication analysis of myeloid cells in HCC. **A** Number and strength of interactions between normal and HCC cell types. **B** Network plot depicting the changes in the number and strength of interactions between normal and HCC cell types. **C** Signaling pathways involved in cell type reception and transmission in normal and HCC cells. **D** Communication strength between myeloid cells and other cell subgroups is characterized by increased or decreased receptors. **E** Receptor pairs associated with both incoming and outgoing signaling pathways of the APP signaling pathway between cells in HCC. **F** Receptor pairs associated with both incoming and outgoing signaling pathways of the MIF signaling pathway between cells in HCC

To gain further insights into the interplay within the TIME of HCC, we investigated the intercellular communication between specific immune subpopulations and myeloid cells involved in ferroptosis. We compared these interactions between HCC tissue and normal tissue to understand the alterations occurring in the cancer context. Compared to normal tissue, we observed an increase in the total number of cell interactions within the HCC microenvironment, while the intensity of these interactions decreased (Fig. 4A). Specifically, the quantity and strength of signals sent from B cells to NK cells increased, while the quantity and intensity of signals sent from NK cells to T cells decreased. Furthermore, the quantity and intensity of signals sent from T cells to myeloid cells increased (Fig. 4B). These patterns of signal reception and transmission between normal tissue and HCC are depicted in Fig. 4C. Notably, the intensity of the GALECTIN signal targeting myeloid cells diminished in HCC, whereas the MHC-I signal originating from myeloid cells strengthened in this context (Fig. 4C).

Considering that myeloid cells represent a highly active subset associated with ferroptosis, we conducted an analysis of receptor-ligand pairs potentially regulating communication between myeloid cells and other immune cells. In HCC, we observed an increased interaction involving the macrophage migration inhibitory factor (MIF) ligand derived from T cells binding to the corresponding receptors on myeloid cells (Fig. 4D). Conversely, the interaction between the amyloid precursor protein (APP) ligand sourced from hepatocytes and the corresponding receptors on myeloid cells decreased in HCC (Fig. 4D). Furthermore, we delved into studying the reciprocal interactions between the MIF and APP pathways within the cellular milieu of HCC. In this context, the APP signaling pathway is emitted from hepatocytes and received by myeloid cells (Fig. 4E), while the MIF signaling pathway is emitted by hepatocytes, plasma cells, NK cells, and T cells, and received by myeloid cells (Fig. 4F). These findings collectively emphasize the complex nature of the TIME in HCC.

### Enrichment analysis of DEGs related to ferroptosis-activated myeloid cells

**Fig. 5:**
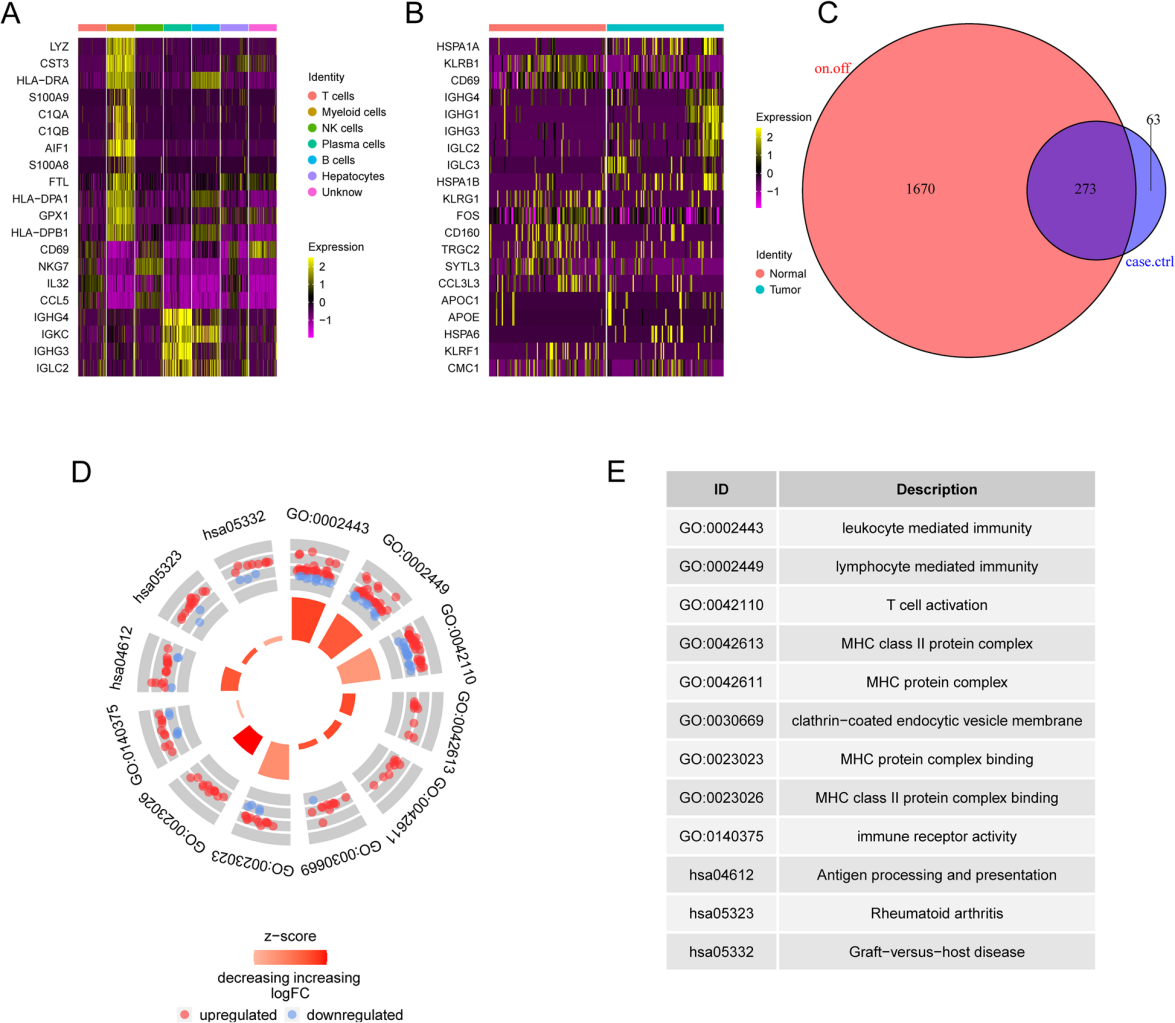
Visualizations of functional enrichment analysis results. **A** Differential expression of genes within ferroptosis-activated myeloid cells in HCC. **B** Significant differential expression of Top 20 DEGs in HCC vs control samples. **C** Venn diagram representing the intersection of key genes. **D** Circular plot illustrating the GO and KEGG enrichment analysis results for the key genes. **E** pathway IDs and corresponding description

To investigate the biological functions and pathways related to differentially expressed genes (DEGs) in ferroptosis-activated myeloid cells, we identified 1943 DEGs by applying statistical criteria. Specifically, we considered genes with significant adjusted *p*-values (< 0.05) and a log2 fold change exceeding a threshold of > 0.25 or <  − 0.25 based on their expression levels, as summarized in Additional file 6: Table S4. To visualize the expression patterns of the top 20 ranked DEGs among these 1943 genes, a heatmap was generated (Fig. 5A). The DEGs included LYZ, CST3, HLA-DRA, S100A9, C1QA, C1QB, AIF1, S100A8, FTL, HLA-DPA1, GPX1, HLA-DPB1, CD69, NKG7, IL32, CCL5, IGHG4, IGKC, IGHG3, and IGLC2. Additionally, by comparing HCC samples to normal controls within the GSE140228 dataset, we identified 336 DEGs that exhibited statistically significant differences between these two groups (Additional file 7: Table S5). Among these DEGs, a heatmap displayed the top 10 upregulated genes (HSPA1A, IGHG4, IGHG1, IGHG3, IGLC2, IGLC3, HSPA1B, APOC1, APOE, and HSPA6) and the top 10 downregulated genes (KLRB1, CD69, KLRG1, FOS, CD160, TRGC2, SYTL3, CCL3L3, KLRF1, and CMC1) in HCC samples (Fig. 5B). Next, we determined the intersection of DEGs between ferroptosis-activated myeloid cells and HCC samples, resulting in a set of 273 hub genes (Fig. 5C). These hub genes represent common differentially expressed genes associated with both ferroptosis activation in myeloid cells and HCC.

The GO analysis results showed enrichment in various biological processes, including leukocyte-or lymphocyte- mediated immunity and T cell activation. In addition, enrichment was observed in several cellular components, such as MHC class II protein complex, MHC protein complex, and clathrin-coated endocytic vesicle membrane (Additional file 8: Table S6). Furthermore, the molecular function analysis revealed enrichment in MHC protein complex binding, MHC class II protein complex binding, and immune receptor activity (Fig. 5D, E). Besides, the enriched KEGG pathways (Additional file 9: Table S7) included antigen processing and presentation, rheumatoid arthritis, and graft-versus-host disease (Fig. 5D, E). Understanding the molecular processes and pathways involved in ferroptosis-activated myeloid cells and their relevance to HCC can help uncover potential therapeutic targets and mechanisms underlying immune responses of patients with HCC.

### Construction and validation of the single-cell ferroptosis risk gene scores

**Fig. 6:**
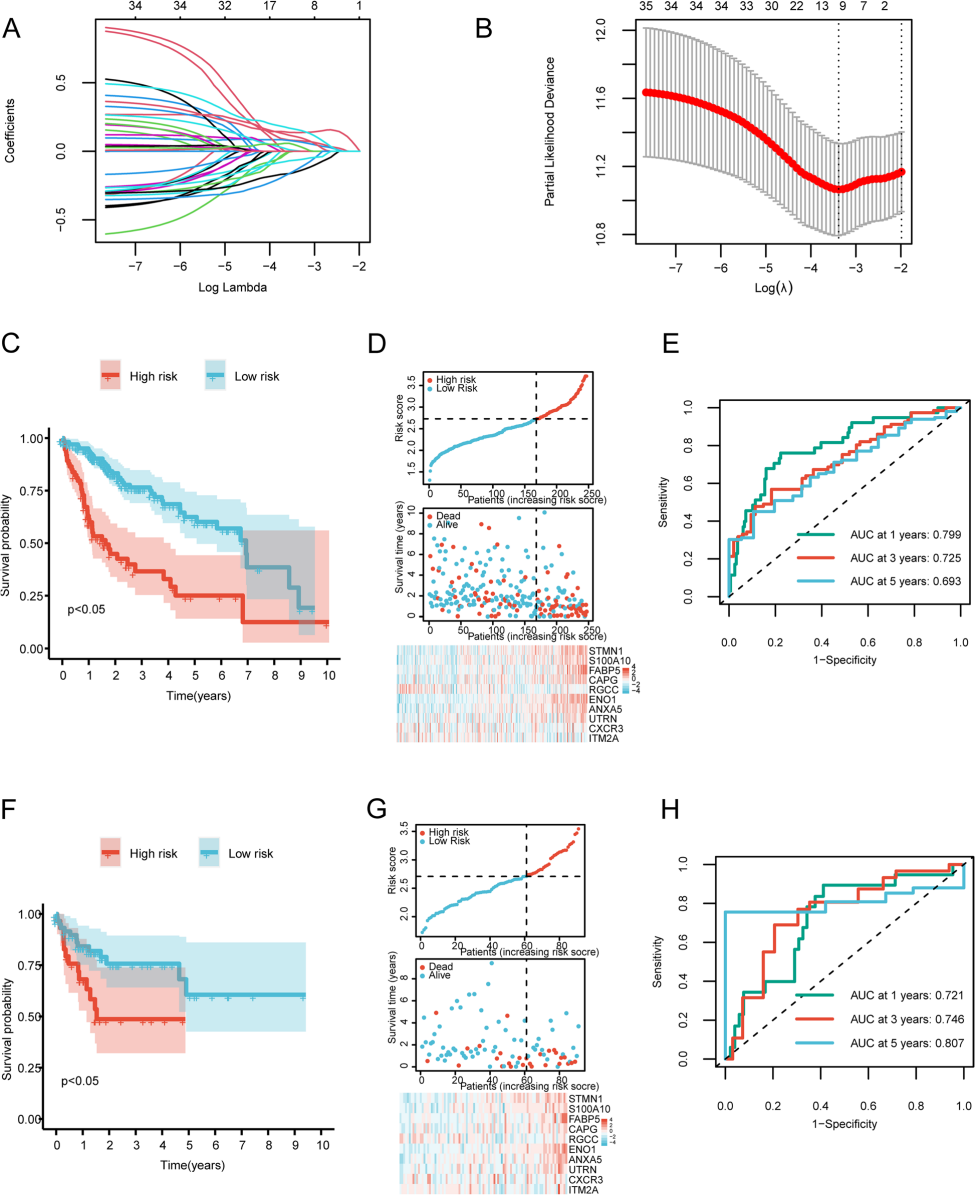
Identification of core genes involved in scFRGs. **A** Trajectory of variable changes in LASSO regression, where the *x*-axis represents the logarithm of the lambda values for the independent variables and the *y*-axis represents the obtainable coefficients. **B** Confidence intervals at each lambda value in LASSO regression. **C** Survival curves of high-risk and low-risk groups of patients from the training cohort. **D** Risk ternary plot of the training set. **E** Time-dependent ROC curves for the 1-year, 3-year, and 5-year models trained on the training cohort. **F** Survival curves of high-risk and low-risk groups of patients from the validation cohort. Red represents the high-risk group, while blue represents the low-risk group. **G** Risk ternary plot of the validation set. **H** Time-dependent ROC curves for the 1-year, 3-year, and 5-year models validated on the validation cohort

To identify genes associated with prognostic-related features within the set of 273 hub genes specific to TCGA-LIHC, we performed univariate Cox analysis. This analysis revealed 36 genes significantly correlated with HCC prognosis, which are summarized in Additional file 10: Table S8. To construct and validate the predictive model, the HCC samples were randomized into two subsets: a training set, which included 7/10 of the samples (*n* = 173), and a validation set, which comprised the remaining 3/10 of the samples (*n* = 91). The training set was subjected to LASSO regression analysis aimed at eliminating redundant genes, leading to the identification of a subset of 10 genes associated with HCC patient prognosis (Additional file 11: Table S9). The selection process is illustrated in Fig. 6A, B. To assess the robustness of the model, we used the calculated median risk score as the threshold to divide the patients into two stratifications based on the 10 gene features. Significant differences in overall survival were observed between the training cohort (Fig. 6C) and the validation cohort (Fig. 6F), with high-risk patients showing significantly worse prognosis compared to low-risk patients across both cohorts according to Kaplan–Meier (KM) survival curves stratified by different age groups, gender, and tumor stages (Additional file 12: Figure S3). The heatmap (Fig. 6D, G) additionally demonstrates the expression patterns of the 10 selected ferroptosis-active genes. Moreover, we assessed the predictive performance of the model using receiver operating characteristic (ROC) curves. In the training queue, the area under the curve (AUC) values for the 1-year, 3-year, and 5-year survival periods were calculated as 0.799, 0.725, and 0.693, respectively (Fig. 6E). In the validation queue, the AUC values for the same time points were 0.721, 0.746, and 0.807, respectively (Fig. 6H). The reliability of the model was further validated using an external cohort of 231 patients from Japan (ICGC-LIRI-JP). Remarkably, consistent with previous findings, the high-risk group exhibited significantly poorer survival outcomes. The ROC curve analysis also substantiated the excellent predictive capacity of our model in this cohort, as depicted in Additional file 13: Figure S4. Hence, the scFRGs scores calculated using the 10 gene features have the potential to predict the prognosis for HCC patients effectively in both groups, indicating their robustness and applicability.

### Distinct pathway patterns between scFRG-related risk subgroups

**Fig. 7:**
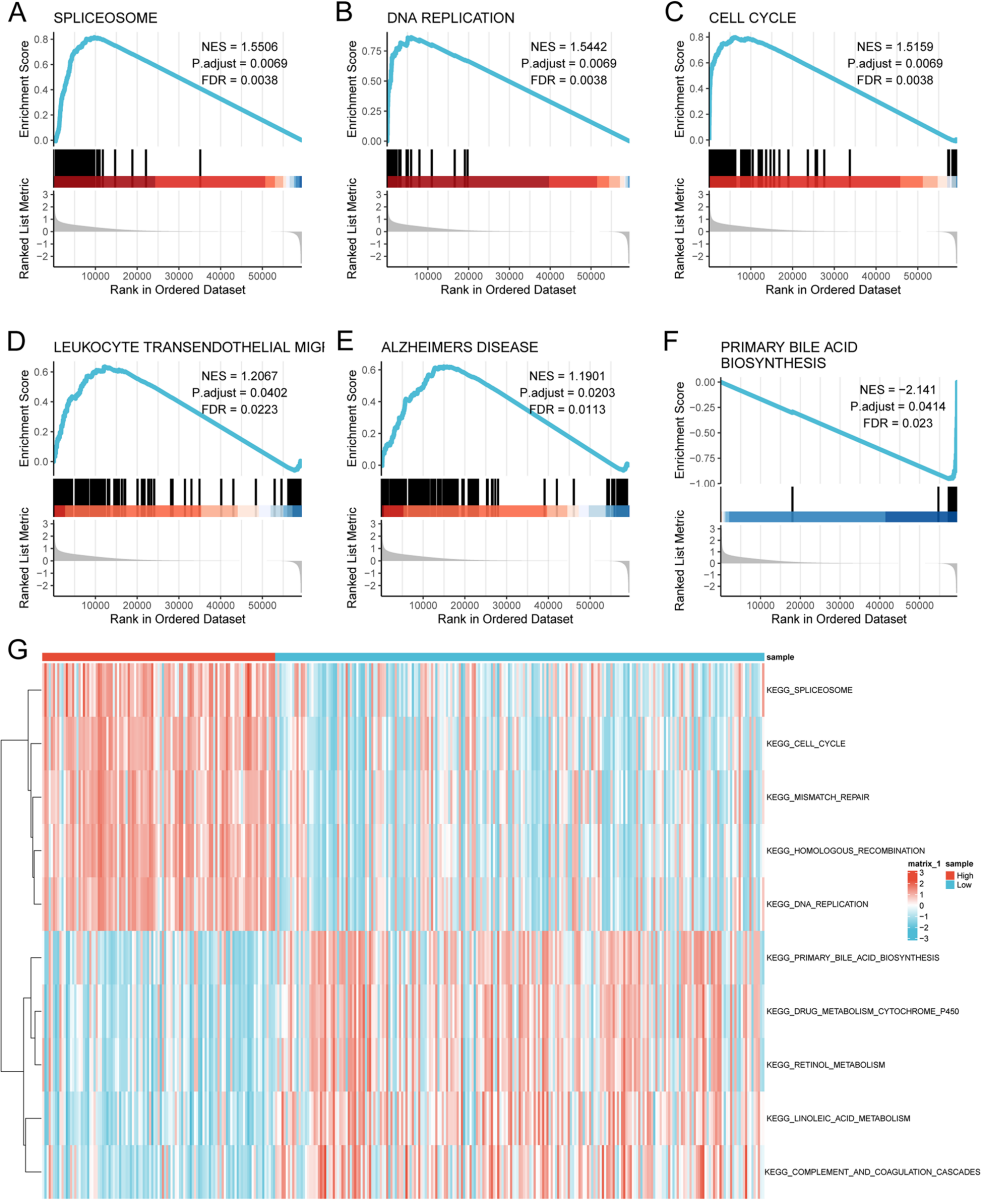
Pathway analysis of the scFRG-related risk subgroups. GSEA analysis reveals the enrichment of the following pathways: **A** SPLICEOSOME, **B** DNA REPLICATION, **C** CELL CYCLE, **D** LEUKOCYTE TRANSENDOTHELIAL MIGR, **E** ALZHEIMERS DISEASE, **F** PRIMARY BILE ACID BIOSYNTHESIS. **G** The pathway heatmap generated from GSVA enrichment analysis depicts the differential enrichment between scFRG-based risk groups

To gain insights into the potential mechanisms underlying the DEGs between scFRG-based risk groups, we conducted GSEA enrichment analysis using pathway information from the MsigDB database. The most significant pathways were identified based on normalized enrichment scores (NES) (Additional file 14: Table S10). The GSEA results indicated significant enrichment in the following pathways: SPLICEOSOME (NES = 1.5506, adjusted *p* = 0.0069, FDR = 0.0038, Fig. 7A), DNA REPLICATION (NES = 1.5442, adjusted *p* = 0.0069, FDR = 0.0038, Fig. 7B), CELL CYCLE (NES = 1.5159, adjusted *p* = 0.0069, FDR = 0.0038, Fig. 7C), LEUKOCYTE TRANSENDOTHELIAL MIGR (NES = 1.2067, adjusted *p* = 0.0402, FDR = 0.0223, Fig. 7D), ALZHEIMERS DISEASE (NES = 1.1901, adjusted *p* = 0.0203, FDR = 0.0113, Fig. 7E), and PRIMARY BILE ACID BIOSYNTHESIS (NES =  − 2.141, adjusted *p* = 0.0414, FDR = 0.0223, Fig. 7F). The most significant differences between risk groups were used to select the top 5 pathways, which were visualized in a pathway activity heatmap (Fig. 7G, Additional file 15: Table S11) based on the function of “GSVA” analysis. Insights into the functional implications of these pathways can shed light on the underlying mechanisms driving HCC and, in turn, facilitate the identification of potential therapeutic targets for developing novel treatment strategies.

### Analysis of immune infiltration and predictive value of the immunotherapy response of scFRG signatures

**Fig. 8:**
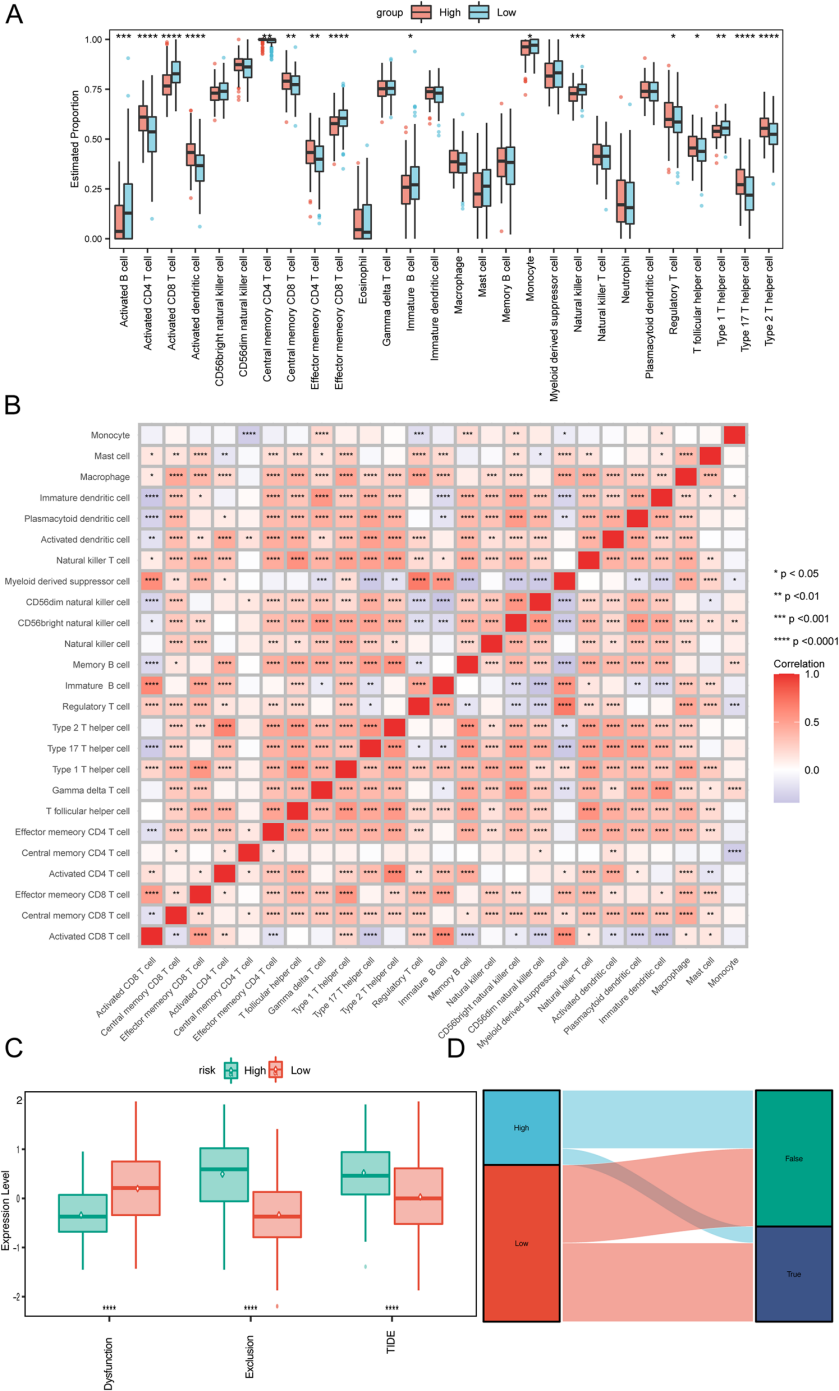
TIME disparities between high- and low-risk groups. **A** Estimated proportions of immune cells between high-risk and low-risk groups. **B** Relationships among immune cells. Asterisks indicate *p*-values: ****p < 0.0001, ***p < 0.001, **p < 0.01, *p < 0.05. **C** Differences in T cell dysfunction, T cell exclusion, and TIDE scores between high-risk and low-risk groups. **D** Predicted response to immune therapy according to TIDE analysis

**Fig. 9:**
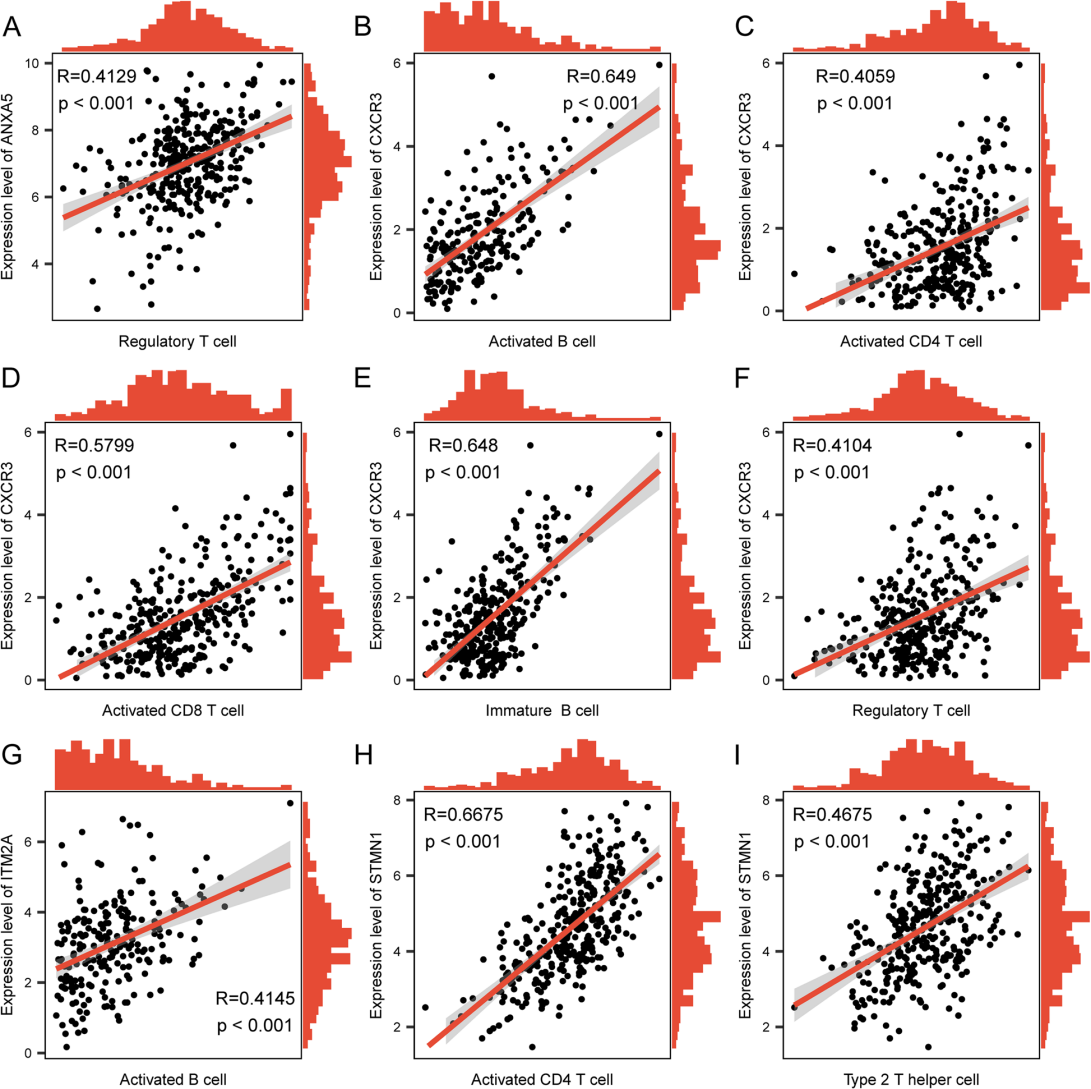
Correlation between ferroptosis-related hub gene and immune cells. **A** Correlation between gene ANXA5 and regulatory T cell. **B**–**F** gene CXCR3 and activated B cell, activated CD4 T cell, activated CD8 T cell, immature B cell, and regulatory T cell. **G** Gene ITM2A and activated B cell. **H**, **I** Gene STMN1 and activated CD4 T cell and type 2 T helper cell illustrated in the scatter plots

To investigate the characteristics in TIME between the high-risk and low-risk groups, we analyzed the infiltration levels of 28 immune cell types (Additional file 16: Table S12) using the ssGSEA method. Significant differences were observed for several immune cells between the groups, including activated B cell, activated CD4 T cell, activated CD8 T cell, activated dendritic cell, and central memory CD4 T cell (*p* < 0.05, Fig. 8A). Most immune cell types showed positive correlations with each other, indicating a coordinated immune response. However, a few immune cell types exhibited negative correlations. Activated CD8 T cells exhibited significant negative correlations with some specific immune cell populations, such as central memory CD8 T cells and effector memory CD4 T cells, as indicated by the blue-colored blocks in Fig. 8B. Furthermore, we found significant correlations between each intersection gene and its corresponding immune cell type (Fig. 9). As for the specific genes, ANXA5 was significantly correlated with regulatory T cell (*R* = 0.4129, *p* < 0.001) (Fig. 9A), while CXCR3 exhibited significant correlations with activated B cell, activated CD4 T cell, activated CD8 T cell, immature B cell, and regulatory T cell (Fig. 9B–F). Additionally, STMN1 showed significant correlations with activated CD4 T cell and type 2 T helper cell (Fig. 9H, I), while ITM2A showed a significant correlation with Activated B cells (Fig. 9G). These findings highlight the associations between specific genes and immune cell types, providing insights into the potential interactions and roles of the immune system within the identified risk subgroups based on scFRGs.

To evaluate the potential response to immunotherapy in various risk subgroups, we utilized the Tumor Immune Dysfunction and Exclusion (TIDE) algorithm (Additional file 17: Table S13). The TIDE prediction score reflects the likelihood of immune evasion, indicating a lower probability of benefiting from immune checkpoint inhibitors (ICIs) treatment. In the conducted investigation, it was observed that the low-risk subgroup demonstrated lower TIDE scores in contrast to the high-risk subgroup. This suggests a lower T cell exclusion score and reduced T cell dysfunction in the high-risk subgroup, as depicted in Fig. 8C. Furthermore, most of the cases showing response to ICIs were observed within the low-risk group (Fig. 8D). This observation suggests that patients with a lower risk score are more likely to derive benefits from ICIs treatment compared to those with a higher risk score. Therefore, understanding the relationships between gene expression and TIME can contribute to a better understanding of the immune landscape in HCC and aid in patient stratification and personalized treatment decisions, including but not limited to immunotherapy.

Furthermore, we plotted time-dependent ROC curves for 1, 3, and 5 years based on the ssGSEA immune infiltration scores of immature B cells and CD4 + T cells. Our findings revealed that the validation efficiency of our model was superior to that of the prognostic models based on the immune infiltration scores of immature B cells and CD4 + T cells in both the training and validation sets of the TCGA-LIHC dataset as well as in the external validation dataset ICGC-LIRI-JP (Additional file 18: Figure S5).

### Tumor mutation burden (TMB) and drug sensitivity analysis

**Fig. 10:**
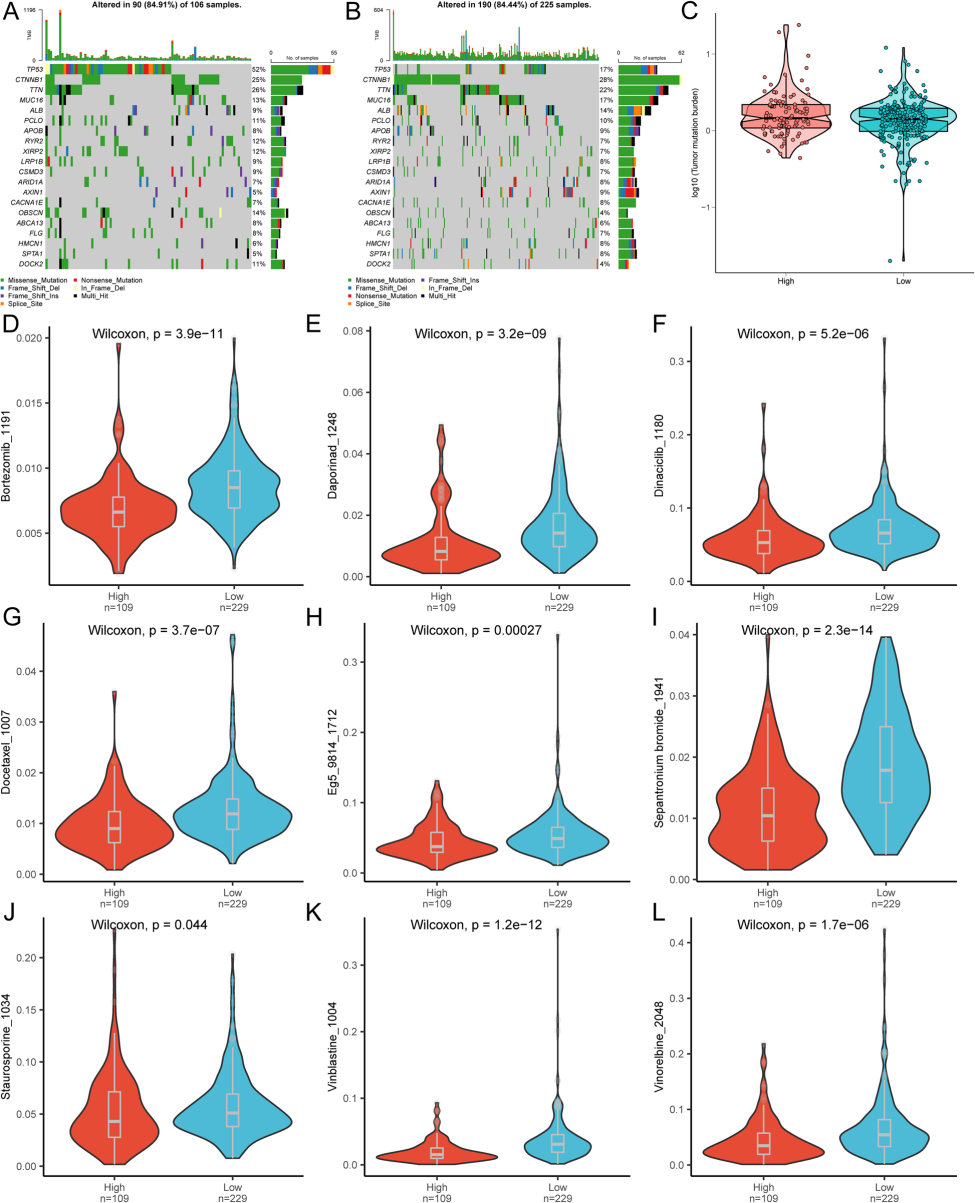
Differences in tumor mutational burden (TMB) and drug sensitivity between high-risk and low-risk groups. Top 20 genes with the highest mutation frequency in the high-risk group (**A**) and the low-risk group (**B**). Difference in tumor mutational burden (TMB) between the high-risk and low-risk groups (**C**). Differential drug sensitivity to Camptothecin_1003 (**D**), CDK9_5038_1709 (**E**), Dactinomycin_1811 (**F**), Dactinomycin_1911 (**G**), Eg5_9814_1712 (**H**), Epirubicin_1511 (**I**), Podophyllotoxin bromide_1825 (**J**), Rapamycin_1084 (**K**), and Podophyllotoxin bromide_1825 (**L**) between the high-risk and low-risk groups

To assess immunotherapy sensitivity in risk-stratified populations with HCC, we evaluated the TMB. Specific gene mutations were detected in HCC, and a visualization was generated for the top 20 mutated genes. Among the analyzed genes, TP53 stood out with the highest mutation frequency in both groups, closely followed by CTNNB1 (Figs. 9B and 10A). Despite the absence of significant differences in TMB between the scFRG-based risk groups (*p* > 0.05) (Fig. 10C), TMB holds promise as a potential marker for predicting immunotherapy response. Considering TMB as an indicative factor sheds light on identifying patients who could potentially benefit from immunotherapy.

Furthermore, as chemotherapy continues to be an effective treatment approach for HCC, we conducted an analysis to determine whether risk scores could reliably predict the response to chemotherapy for patients with HCC. We examined the response to various drugs such as Bortezomib_1191, Daporinad_1248, Dinaciclib_1180, Docetaxel_1007, Eg5_9814_1712, Sepantronium bromide_1941, Staurosporine_1034, Vinblastine_1004, and Vinorelbine_2048 (Additional file 19: Table S14). Based on our analysis, it was observed that patients with high-risk scores may have a higher likelihood of positive response to Bortezomib_1191, Daporinad_1248, Dinaciclib_1180, Docetaxel_1007, Eg5_9814_1712, Sepantronium bromide_1941, Staurosporine_1034, Vinblastine_1004, and Vinorelbine_2048 (Fig. 10D–L). These findings underscore the potential of chemotherapy as a promising treatment option for the high-risk group. They also emphasize the significance of risk scores in predicting both immunotherapy response (via assessment of TMB) and chemosensitivity in HCC. By integrating risk stratification with mutational analysis and drug sensitivity profiling, personalized treatment strategies can be developed, facilitating more effective and tailored approaches for patients based on their individual risk profiles.

### Prognostic nomogram for scFRG-based risk model in HCC

**Fig. 11:**
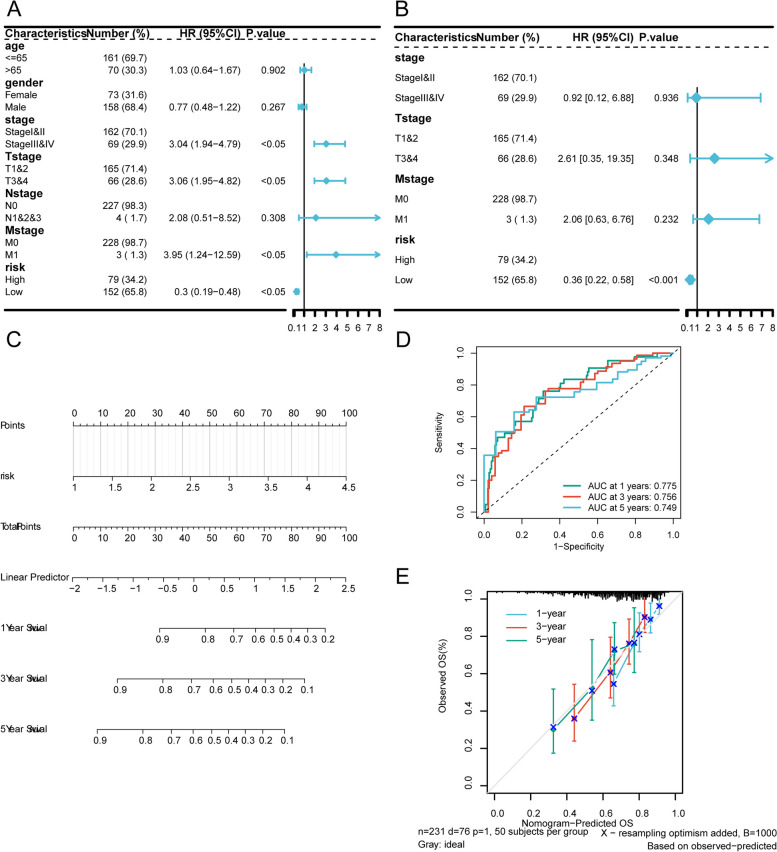
Nomogram construction with risk score and clinical characteristics. Univariate Cox regression (**A**) and multivariate Cox regression (**B**) analysis on clinical features displayed on forest plot. **C** Nomogram of the predictive model. Each segment represents the contribution of a clinical factor to the outcome event, and the total score represents the sum of individual scores for all variable values. The bottom three lines represent the prognosis corresponding to 1-year, 3-year, and 5-year survival periods for each value point. Time-dependent ROC curves (**D**) and calibration curves (**E**) for the 1-year, 3-year, and 5-year of scFRGs model

The potential of the scFRG-based risk model was validated through univariate and multivariate Cox regression analyses on clinicopathological characteristics, revealing a consistent independent prognostic risk factor for HCC patients (Fig. 10B and 11A). A predictive nomogram was established from the multivariate Cox regression results (Fig. 11C), visually illustrating each variable’s contribution to overall prognosis and enabling survival probability estimation at specific time points. Performance assessment of the risk model involved generating ROC curves; AUC values of 0.775, 0.756, and 0.749 were obtained for 1-year, 3-year, and 5-year survival rates respectively (Fig. 11D). Moreover, a calibration curve displayed strong agreement between predicted and observed 1/3/5-year survival probabilities (Fig. 11E). As a practical tool, the nomogram aids clinicians in estimating individual patient prognosis based on multiple variables, thereby augmenting personalized treatment decision-making and patient management.

### Validation of differential expression of ferroptosis-related signature genes in transcription and protein levels

**Fig. 12:**
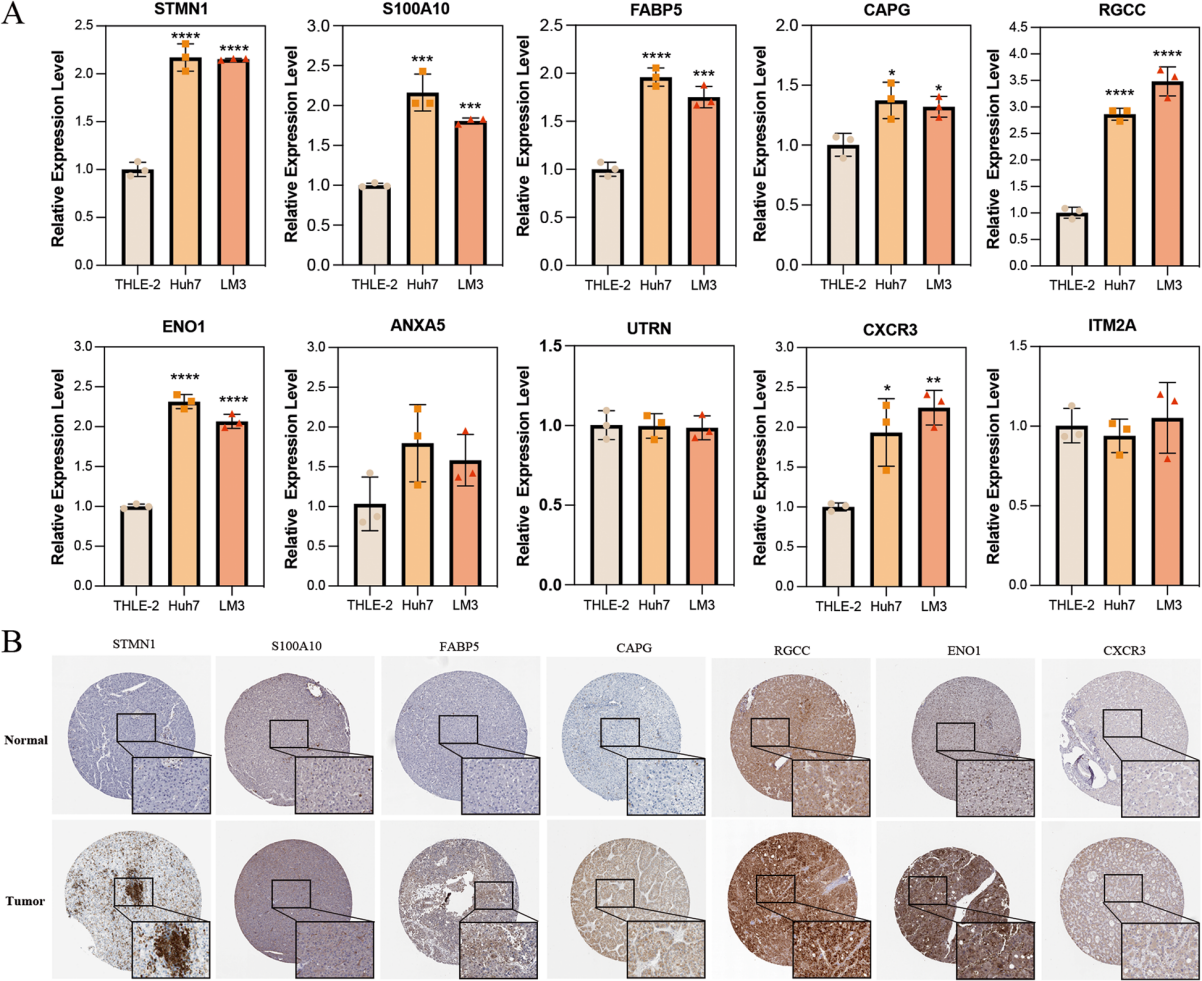
Verification at the transcriptional and protein levels of ferroptosis-related genes. Expression of 10 genes in different cell lines (**A**). Protein of significant genes in HCC tumor tissues compared to normal tissues (**B**). Data are shown as the mean ± SD of three experiments, two-tailed Student’s *t* test: ns, nonsignificant (*p* > 0.05), *p < 0.05, **p < 0.01, ***p < 0.001, ****p < 0.0001

To further verify the expression differences of the selected 10 signature genes related to ferroptosis activity in tumor and normal cells, we performed qRT-PCR analysis to assess their expression levels in human hepatocellular carcinoma cell lines Huh7 and LM3 relative to normal liver cells THLE-2 (Fig. 12A). The study confirmed an elevated trend in the transcription levels of these 10 genes in both Huh7 and LM3. Notably, seven genes (STMN1, S100A10, FABP5, CAPG, RGCC, ENO1, CXCR3) exhibited significant statistical differences compared to THLE-2 (*p* < 0.05). Additionally, we validated the protein-level expression of these seven genes through immunohistochemistry using the Human Protein Atlas (HPA), which substantiated their high expression in tumor tissues (Fig. 12B).

## Discussion

Hepatocellular carcinoma (HCC) is a prevalent and aggressive tumor associated with high morbidity rates among patients. While certain environmental and genetic risk factors have been identified in relation to HCC, the underlying molecular processes leading to its development remain largely unknown. Sorafenib has emerged as the primary targeted therapy for advanced HCC. Inducing ferroptosis and enhancing its sensitivity while circumventing apoptosis in sorafenib resistance could effectively enhance the treatment outcomes for HCC, reducing the occurrence of drug resistance [14]. Drug insensitivity or resistance presents significant challenges in the management of HCC, highlighting the need for therapeutic agents with a distinct mechanism of action. Identifying such agents is crucial to overcoming these obstacles and developing new treatment strategies that effectively target HCC, ultimately improving patient outcomes [15].

Ferroptosis, defined by iron-dependent lipid peroxidation, epitomizes an exceptional cellular demise modality that distinguishes itself from programmed cell death mechanisms like apoptosis [16]. Focusing on ferroptosis emerges as an innovative strategy to combat HCC [17]. The synergistic utilization of ferroptosis modulators with chemotherapy or ICIs represents a highly promising therapeutic strategy, despite the ongoing laboratory-stage studies [11]. Mechanistically, by regulating ferroptosis, it is possible to enhance the sensitivity of HCC cell lines and reshape the immunosuppressive microenvironment in HCC, ultimately transforming “cold” tumors into “hot” tumors that respond better to treatment [18–20]. Consequently, there is an increasing demand for robust ferroptosis-related gene (FRG) signatures that accurately predict patient outcomes in HCC. However, current predictive models lack single-cell-level precision. In our study, we utilize single-cell sequencing technology and machine learning to fill this gap. By leveraging single-cell sequencing and machine learning, we identify robust FRG signatures as reliable prognostic tools in HCC. This approach allows us to distinguish tumor cells from normal cells based on ferroptosis-active cell subpopulations. Furthermore, we perform pseudotemporal analysis and cell communication analysis on these specific subgroups, providing deeper insights into the biological background and functional interaction networks of the ferroptosis model.

To validate our findings, we extensively validate and predict prognostic outcomes using external datasets, strengthening the reliability of our results. Additionally, we thoroughly examine genomic variations, drug sensitivity differences, and immune infiltration characteristics between high and low-risk groups, offering a multidimensional understanding of the clinical implications of our model.

These comprehensive analyses provide nuanced insights into the clinical implications of our model, aiding in personalized patient management.

This single-cell ferroptosis-based classification system aims to categorize HCC tumors based on their molecular characteristics related to ferroptosis. *STMN1*, *S100A10*, *FABP5*, *CAPG*, *RGCC*, *ENO1*, *ANXA5*, *UTRN*, *CXCR3*, and *ITM2A*, a set of ten scFRGs signatures, have been adopted for acquiring a refined model by LASSO-Cox regression approach. Upregulation of *STMN1* in HCC facilitates microvascular infiltration and metastasis, is associated with immune infiltration and DNA methylation alterations, and serves as an independent prognostic factor [21, 22]. S100A10, belonging to the S100 family, plays a crucial role in HCC stemness-related properties, making it a potential diagnostic marker for HCC progression [23]. FABP5, identified as an immunometabolic marker in HCC, correlates with improved overall survival and CD8 + T cell infiltration [24]. Elevated CAPG expression in tumors indicates a poorer prognosis in breast cancer patients [25]. RGC-32 serves as a marker for M2 macrophage polarization and influences the immune response within the tumor microenvironment [26, 27]. Additionally, abnormal expression of α-enolase 1 (ENO1) inhibits ferroptosis in HCC cells by degrading iron regulatory protein 1 mRNA [28]. Overexpression of ANXA5 enhances clinical progression and lymphatic metastasis in HCC patients [29]. In melanoma, reduced expression of UTRN is associated with advanced clinical characteristics and poorer prognosis, leading to shorter survival time [30]. Meanwhile, CXCR3, an important chemokine receptor, is a key mediator of crosstalk between myeloid and B cells, which helps to shape the microenvironments of primary and secondary HCC [31]. ITM2A was identified as a hub mRNA prognostic biomarker within a competitive endogenous RNA (ceRNA) regulatory network in HCC [32]. In line with the aforementioned findings, our study provides additional experimental evidence to confirm the significant upregulation of seven genes (*STMN1*, *S100A10*, *FABP5*, *CAPG*, *RGCC*, *ENO1*, and *CXCR*) in hepatocellular carcinoma cell lines. Furthermore, immunohistochemistry results further bolster these conclusions, underscoring the robust biological foundation of our model. These findings serve as a crucial cornerstone for advancing comprehensive basic research and facilitating the clinical translation of our model.

The scFRG-based risk score model for HCC was developed combined machine learning techniques and gene expression features. The formula consists of weights assigned to specific genes and was validated as an independent prognostic factor for HCC. A nomogram model based on these ten genes was constructed, providing an intuitive prediction of HCC patients’ overall survival at 1, 3, and 5 years. The accuracy of these genes was demonstrated in both the training and validation sets using the TCGA-LIHC dataset. Notably, the low-risk score group exhibited significantly longer overall survival compared to the high-risk score group. Mutations in TP53, MUC16, and TTN were found to be frequently associated with poor prognosis in various cancers, consistent with previous research [33–35]. Differences in the activation levels of immune cell populations were observed between the high- and low-risk groups, suggesting that the risk score not only correlates with prognosis but also reflects differences in the immune response within the tumor microenvironment. Our study revealed significant correlations between ANXA5, CXCR3, ITM2A, and STMN1 genes among the scFRGs and specific immune cell populations, providing valuable insights into the potential role of these genes in modulating the immune response in HCC.

In addition to our study on risk score development and gene expression analysis, pathway enrichment analysis was conducted to delve deeper into the biological implications of the identified signatures. The identification of significant enrichment of intersecting genes in pathways associated with immune response, specifically pertaining to leukocyte-mediated immune processes such as lymphocyte-mediated immunity and T cell activation, suggests a close correlation between ferroptosis in HCC and the TIME. Dysregulation of immune responses and the interplay between iron metabolism, oxidative stress, and immune system function could play a role in the development and progression of HCC. It is noteworthy that based on the TIDE prediction, the low-risk group identified by scFRGs may have a higher likelihood of benefiting from immunotherapy. Interestingly, based on IC50 predictions, individuals at high risk may indeed benefit from chemotherapy treatments such as docetaxel, vinblastine, and vinorelbine. This finding further emphasizes the importance of scFRGs as potential prognostic factors and immune-related gene sets in guiding personalized treatment strategies for hepatocellular carcinoma patients.

## Conclusions

In conclusion, our study has provided significant insights into the prognostic implications, molecular characteristics, immunological factors, and pharmacogenomic aspects related to ferroptosis in HCC at single-cell resolution. Our findings highlight the potential of targeting ferroptotic cell death as a therapeutic strategy and prognostic indicator in HCC. The development of a novel ferroptosis classification system using ten scFRG signatures holds promise for accurately predicting survival outcomes in HCC patients. By integrating gene expression features, we have successfully constructed an independent risk score formula and developed a nomogram model for overall survival prediction. Moreover, our analysis has revealed distinct mutational profiles and variations in the tumor immune microenvironment among scFRG-based risk groups, providing insights into the intricate interplay between ferroptosis, immune response, and HCC progression. These findings shed light on the complex molecular mechanisms underlying ferroptosis and immune response in HCC. Further research is warranted to gain a comprehensive understanding of the identified genes and pathways in HCC and explore their therapeutic implications. Such endeavors will contribute to advancing our knowledge and translating these models into clinical applications, ultimately improving patient management and outcomes in HCC.

## Methods

### Bulk transcriptome data curation

The HCC’s whole-genome transcriptomic profiles, clinical information, and single-nucleotide variation (SNV) data were primarily obtained via the TCGAbiolinks package from the TCGA (https://portal.gdc.cancer.gov/). A total of 374 HCC samples and 50 normal control tissues were included, amounting to 424 cases. Among these, 338 HCC samples with survival information were used for survival-related analysis. Inter-group differential analysis of TCGA-LIHC transcriptomic data was conducted utilizing the R package limma (version 3.50.0) [36]. The validation data were mainly from the ICGC database (https://dcc.icgc.org), and the ICGC-LIRI-JP included a total of 231 primary HCC samples with complete prognostic information.

### Single-cell RNA sequencing data curation

The single-cell dataset GSE140228 was manually downloaded from the GEO (http://www.ncbi.nlm.nih.gov/geo) database and imported for analysis using the Seurat package (version 4.2.0) [37]. In this study, we obtained a cohort consisting of 8 HCC samples and 5 normal tissues for analysis purposes. Only cells with gene expression counts fluctuating between 200 and 60,000 were included for analysis, and the percentage of mitochondrial genes was restricted to below 10%. Single-cell gene expression profile was preprocessed and subjected to principal component analysis (PCA) for dimensionality reduction. Subsequently, the cells were clustered using the Louvain algorithm on the K-nearest neighbors (KNN) graph, constructed based on the principal component (PC) space. The batch effects across different samples were mitigated using the Harmony method [38]. A total of 17 clusters were generated with 30 PC components at a resolution of 0.5 using t-SNE in Seurat. We utilized the FindAllMarkers function with default parameters to identify differentially expressed genes (DEGs) within the cell clusters. Subsequently, we employed cell type-specific biomarkers to classify the cell clusters and further quantified and evaluated the proportions of different cell types.

### Ferroptosis-related gene score based on single-cell clusters

AUCell is a statistical method used specifically for single-cell analysis to determine if a given gene set is enriched at the top quantile of a ranked gene feature [39]. In this study, we utilized AUCell package (version 1.18.1) to calculate the enrichment scores by determining the AUC for a selected set of 484 ferroptosis-related genes. Cells exhibiting greater gene expression within the given gene set exhibited higher AUC values. The “AUCell exploreThresholds” function was used to determine the threshold for identifying cells with gene set activity. Finally, the AUC score of each cell was visualized on the t-SNE embedding using the ggplot2 R package to identify clusters with active gene sets.

### Pseudotime trajectory analysis

The Monocle2 algorithm was utilized for conducting developmental trajectory analysis using genes with high dispersion and expression levels (dispersion estimate ≥ 1 and mean expression ≥ 0.1) [40, 41]. Genes exhibiting significant expression variation along the branches were chosen for further Branch Expression Analysis Modeling (BEAM) [41].

### Cell-communication analysis

The “CellChat” R package is used to explore cellular communication and molecular mechanisms between single cells [42]. The “mergeCellChat” function is employed to combine each group’s CellChat objects, enabling comparisons of the total number of interactions and interaction strengths. Additionally, the “netVisual” series of functions are utilized for visualization purposes.

### Pathway enrichment analyses

The “clusterProfiler” R package (version 4.2.2) was utilized to perform GO and KEGG enrichment analyses to explore the biological functions and pathways of differential gene sets in cell subpopulations exhibiting high ferroptotic activity in hepatocellular carcinoma [43–45]. To investigate the potential functions linked to highly correlated DEGs, we conducted GSEA utilizing molecular signatures c2 from MSigDB database (http://software.broadinstitute.org/gsea/msigdb). For each analysis, gene set permutations were performed 1000 times based on an ordered list of all genes according to their log2FC values [46]. Significantly enriched gene sets were identified based on a *p*-value < 0.05. Additionally, we employed GSVA and visualized the outcomes using the “pheatmap” R package (version 1.0.12) [47].

### Construction and evaluation of the scFRGs prognostic model

To assess the correlation between differentially expressed genes related to ferroptosis at the single-cell level and overall survival (OS) in the tumor cohort, we combined the data from TCGA-LIHC and randomly divided it into a training set and a validation set in a 7:3 ratio. We performed univariate Cox regression analysis to evaluate the association of each gene with OS. Genes with a *p*-value < 0.05 were included in the LASSO Cox regression model, and the penalty parameter (*λ*) was determined based on the minimum criteria [48]. By applying the risk formula, we performed calculations to assign scores for individual patient and subsequently classified them into different risk stratifications. We assessed translational value by analyzing the risk model alongside clinicopathological features using univariate and multivariate Cox regression. Additionally, we employed the “RMS” package in R to construct a validated nomogram for predicting overall survival rates at multiple time intervals. Similarly, we employed the same method to conduct external validation using data from the ICGC-LIRI-JP.

### Immune infiltration, TMB, and immune therapy response within the scFRGs model

#### Immune cell infiltration

We downloaded immune cell expression profile data from the TISIDB (Tumor and Immune System Interactions Database) (http://cis.hku.hk/TISIDB/index.php) as a reference. Using the ssGSEA function in the GSVA package, we quantitatively analyzed the expression profiles of 28 immune cell types based on cancer samples from TCGA-LIHC [24].

### TMB

The “maftools” package was employed to curate the SNV data from TCGA-LIHC, enabling us to visualize somatic variations such as single-nucleotide polymorphisms (SNPs), insertions and deletions (INDELs), TMB, and mutation frequency [49].

### TIDE

To evaluate the response to immune therapy, we performed TIDE (Tumor Immune Dysfunction and Exclusion, http://tide.dfci.harvard.edu/) analysis [50].

To compare the superiority of the prognostic model based on the ferroptosis signature with that based on immune cells, we used data from TCGA-LIHC and ICGC-JP to plot time-dependent ROC curves for 1, 3, and 5 years based on the ssGSEA immune infiltration scores of immature B cells and CD4 + T cells.

### Drug sensitivity analysis

To analyze drug sensitivity, we obtained the half-maximal inhibitory concentration (IC50) data and corresponding gene expression data from the GDSC database (Genomics of Drug Sensitivity in Cancer, https://www.cancerrxgene.org/) [51]. Using the “oncoPredict” R package (version 0.2), we predicted the potential therapeutic drug sensitivity in high and low-risk groups of TCGA-LIHC cancer patients [52].

### Cellular level quantitative real-time polymerase chain reaction (qRT-PCR)

Total RNA was extracted from cells using RNAex Pro (AG, Hunan, China). The quality of total RNA was assessed using a spectrophotometer (Thermo Scientific™ NanoDrop™ One). Reverse transcription was carried out using Evo M-MLV reverse transcriptase (AG) on a TaKaRa PCR Thermal Cycler. The reaction conditions included incubation at 37 °C for 15 min, followed by denaturation at 85 °C for 5 s and cooling to 4 °C for 10 min. For qRT-PCR, gene expression analysis was performed on a LightCycler 96 (Roche) PCR System using SYBR Green Pro Taq HS (AG). The reaction conditions involved a preincubation step at 95 °C for 600 s, followed by 40 cycles of denaturation at 95 °C for 10 s, annealing at 59 °C for 10 s, and extension at 72 °C for 10 s. Relative gene expression levels were calculated using the 2^−ΔΔCt^ method. The primer sequences are provided in Additional file 20: Table S15. Protein expression levels were evaluated through immunohistochemistry analysis obtained from the Human Protein Atlas (HPA). Detailed information can be found on the website http://www.proteinatlas.org/.

### Statistical analysis

Continuous variables were examined using the non-parametric Wilcoxon test, while proportions were compared using either the chi-square test or Fisher’s exact test. Kaplan–Meier survival curves were fitted using the “ggsurvplot” function from the “survminer” package in R software, and statistical differences were assessed using the log-rank test. Feature gene selection and model construction were carried out using LASSO-Cox regression analysis, with predictive performance evaluated through ROC and time-dependent ROC curves. A significance level of *p* < 0.05 (two-tailed) indicated statistical significance. All analyses were conducted using R software version 4.1.2.

### Supplementary Information

Additional file 1: Table S1. Gene list of ferroptosis-associated genes.

Additional file 2: Table S2. Cell List of 585 newly defined activated cells in ferroptosis.

Additional file 3: Figure S1. Expression profile of top four genes in state within cellfate1.

Additional file 4: Figure S2. Expression profile of top four genes in state within cellfate2.

Additional file 5: Table S3. Enriched GO pathways for different gene clusters on ferroptosis-activated myeloid cells.

Additional file 6: Table S4. Differential gene list based on specific cell types.

Additional file 7: Table S5. Differential gene expression profile between hepatocellular carcinoma and normal control group.

Additional file8 : Table S6. GO Enrichment analysis terms utilizing intersection of key genes.

Additional file 9: Table S7. KEEG pathways terms utilizing intersection of key genes.

Additional file 10: Table S8. Gene list with prognostic significance for hepatocellular carcinoma revealed by Cox regression analysis.

Additional file 11: Table S9. 10 genes associated with HCC patient prognostic model.

Additional file 12: Figure S3. Kaplan-Meier (KM) survival curves stratified by different age groups (A & B), gender (C & D), and tumor stages (E-H).

Additional file 13: Figure S4. Validation of model predictive capacity and survival outcomes in Japanese cohort (ICGC-LIRI-JP) using ROC curve analysis.

Additional file 14: Table S10. GSEA enrichment analysis underlying DEGs in scFRG-based risk groups.

Additional file 15: Table S11. GSVA enrichment analysis terms between scFRG-based risk groups.

Additional file 16: Table S12. Immune cell signature list.

Additional file 17: Table S13. Results of TIDE prediction for immunotherapy response in various risk subgroups.

Additional file 18: Figure S5. Internal and external dataset validation of ROC curves for immune cells at 1, 3, and 5 Years. (A) Immature B cell, (B) Activated CD4 T cell, (C) Central memory CD4 T cell, (D) Effector memory CD4 T cell.

Additional file 19: Table S14. Prediction of chemotherapy response in HCC patients based on risk scores.

Additional file 20: Table S15. Table for the primer sequences used in qRT-PCR.

Additional file 21: Retrieval of the external validation dataset ICGC-LIHC-JP from the ICGC official website.

Additional file22: Data Analysis of Bulk RNA and Single-Cell Sequencing.

## Data Availability

All data and code generated or analyzed in this study are included in this published article, its supplementary information files, or publicly accessible repositories. Any additional information needed to reanalyze the data reported in this study can be obtained upon request from the corresponding author. TCGA (https:// www.cancer.gov/tcga), GSE140228(https://www.ncbi.nlm.nih.gov/geo/query/acc.cgi?acc=GSE140228). ICGC data can be found as supplementary material in additional file 21. Code used for analysis can be found in additional file 22.

## Abbreviations

HCC: Hepatocellular carcinoma
scFRGs: Single-cell ferroptosis-related genes
TMB: Tumor mutation burden
TIME: Tumor immune microenvironment
SNV: Single-nucleotide variation
GEO: Gene Expression Omnibus
TCGA: The Cancer Genome Atlas
PCA: Principal component analysis
DEGs: Differentially expressed genes
AUC: Area under the recovery curve
GO: Gene Ontology
KEGG: Kyoto Encyclopedia of Genes and Genomes
GSEA: Gene Set Enrichment Analysis
ssGSEA: Single-sample Gene Set Enrichment Analysis
GSVA: Gene Set Variation Analysis
OS: Overall survival
ROC: Receiver operating characteristic curves
TIDE: Tumor Immune Dysfunction and Exclusion
MIF: Macrophage migration inhibitory factor
APP: Amyloid precursor protein
ICIs: Immune checkpoint inhibitors
