# Supplementary figures and images for: A novel single-cell model reveals ferroptosis-associated biomarkers for individualized therapy and prognostic prediction in hepatocellular carcinoma
Source: BMC Biol. 2024 Jun 10;22:133. doi: 10.1186/s12915-024-01931-z (PMC11163722; doi:10.1186/s12915-024-01931-z)

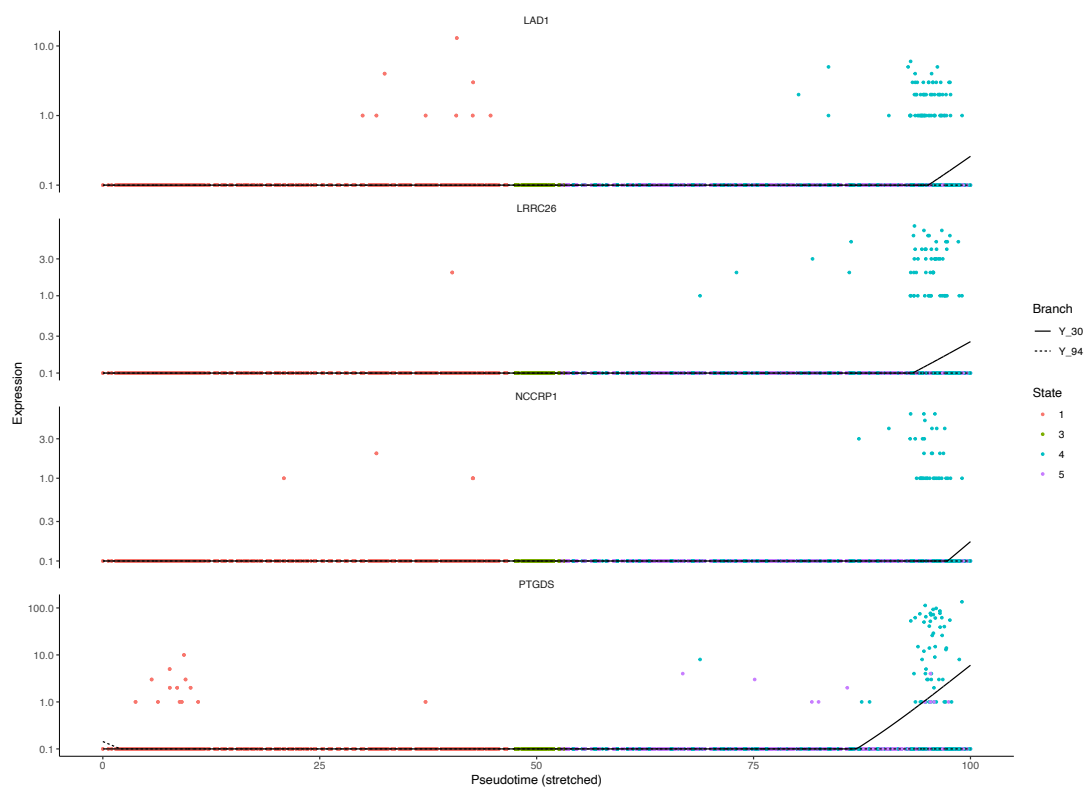

Figure S1. Expression profile of top four genes in state within cellfate1

Supplement: Supplementary file 3 — Additional file 3: Figure S1. Expression profile of top four genes in state within cellfate1. [file 12915_2024_1931_MOESM3_ESM.pdf]

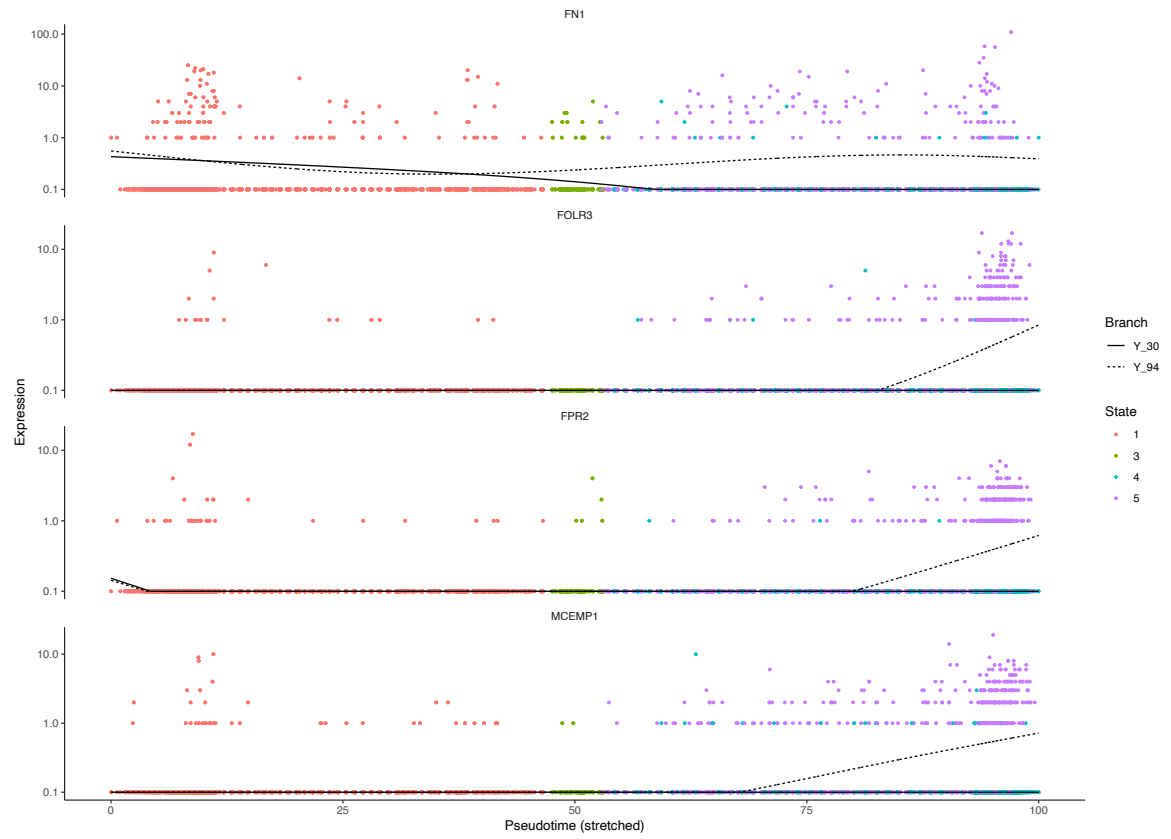

Figure S2. Expression profile of top four genes in state within cellfate2

Supplement: Supplementary file 4 — Additional file 4: Figure S2. Expression profile of top four genes in state within cellfate2. [file 12915_2024_1931_MOESM4_ESM.pdf]

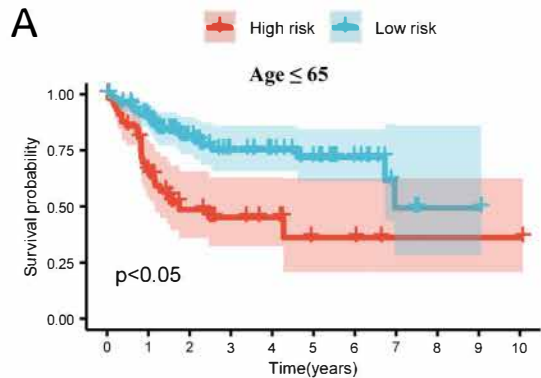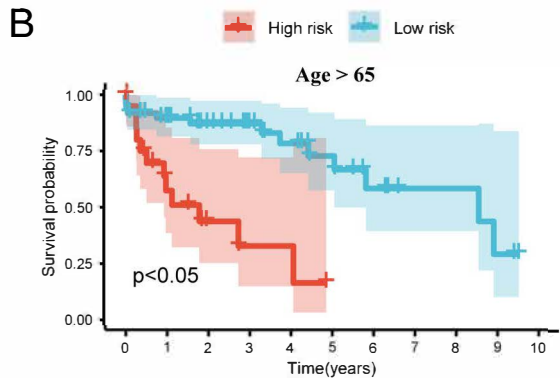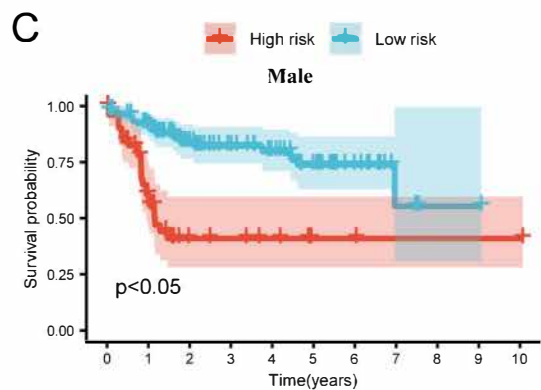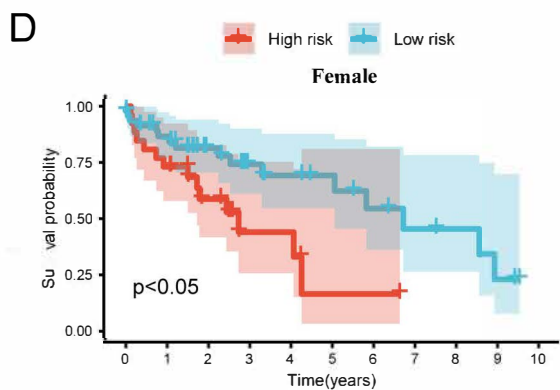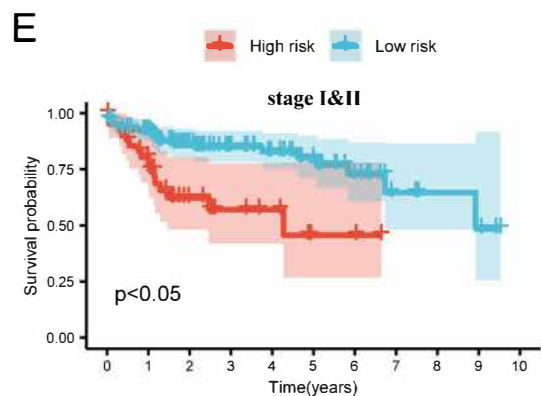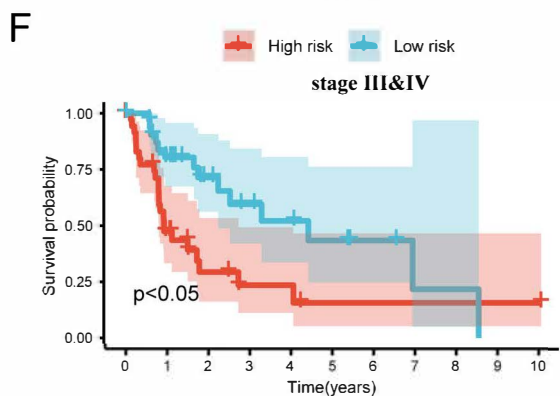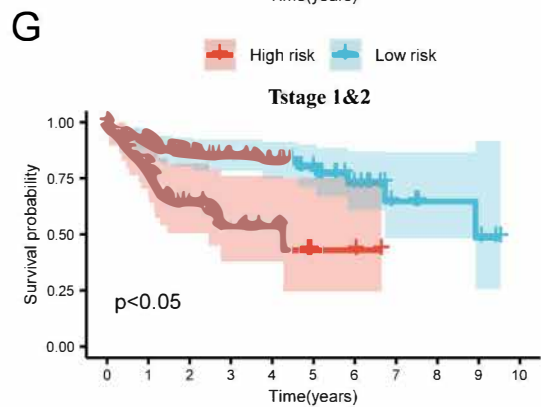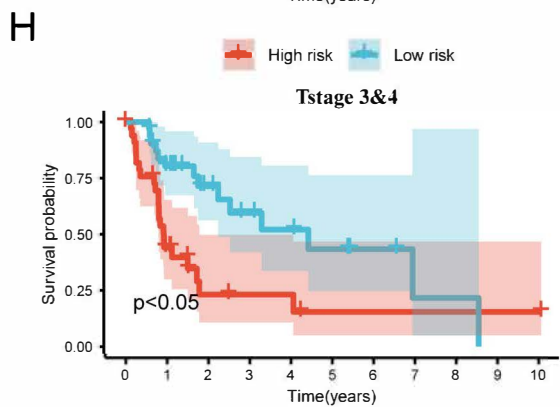

Supplement: Supplementary file 12 — Additional file 12: Figure S3. Kaplan-Meier (KM) survival curves stratified by different age groups (A & B), gender (C & D), and tumor stages (E-H). [file 12915_2024_1931_MOESM12_ESM.pdf]

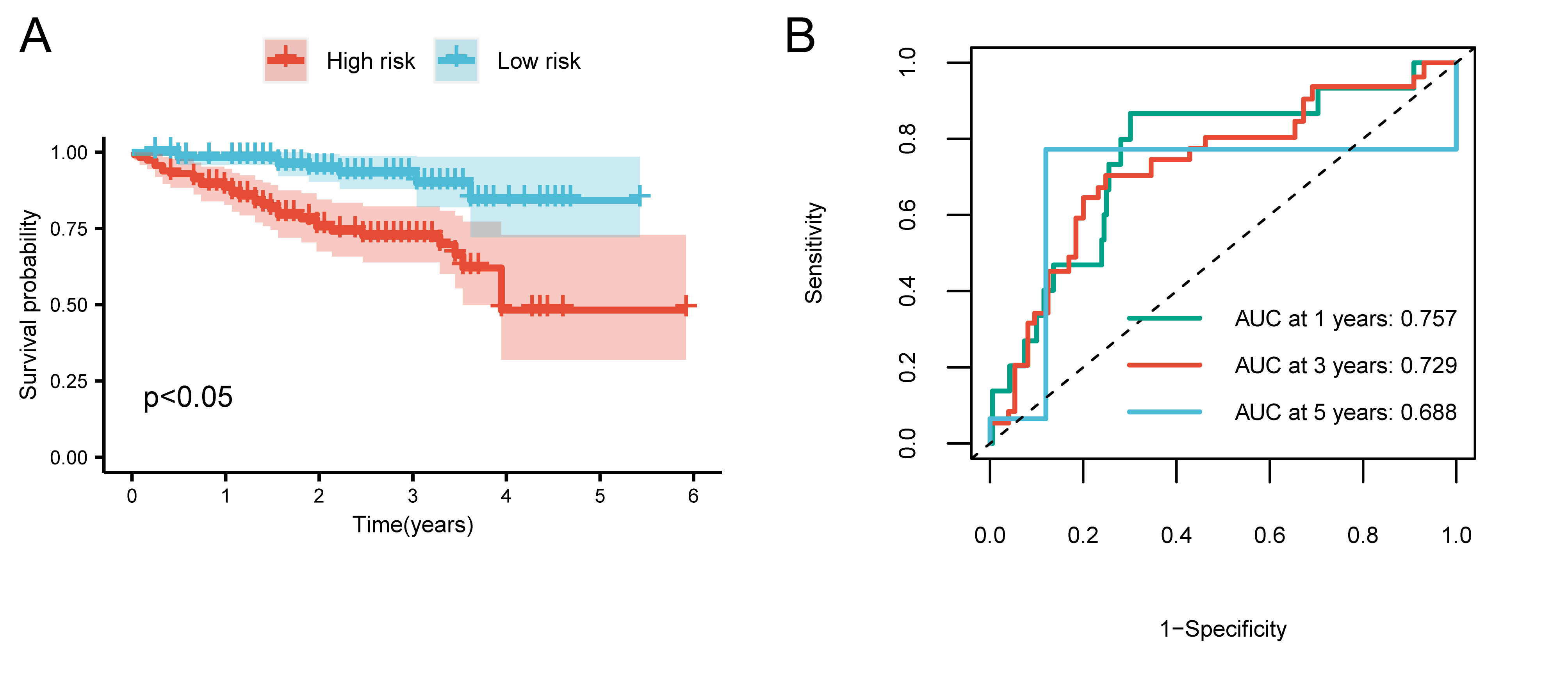

Supplement: Supplementary file 13 — Additional file 13: Figure S4. Validation of model predictive capacity and survival outcomes in Japanese cohort (ICGC-LIRI-JP) using ROC curve analysis. [file 12915_2024_1931_MOESM13_ESM.tif]

**A**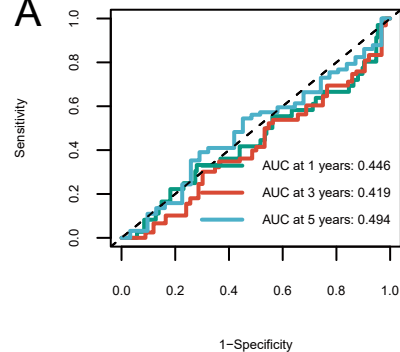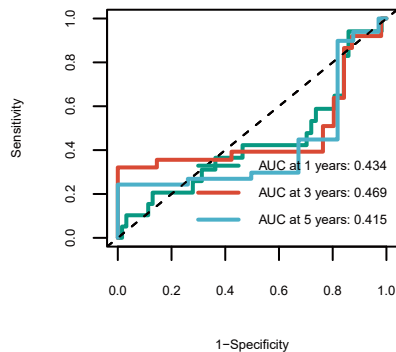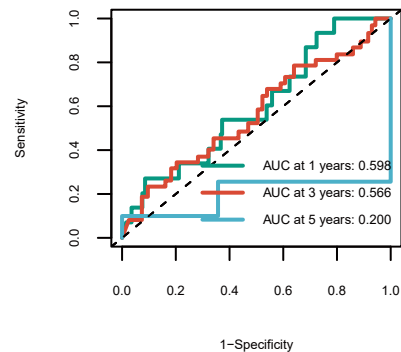**B**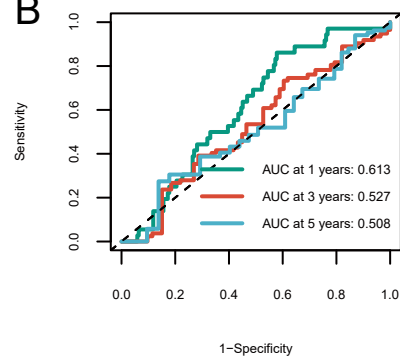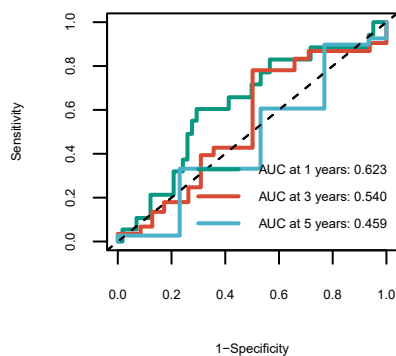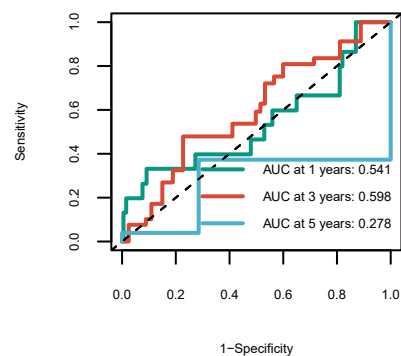**C**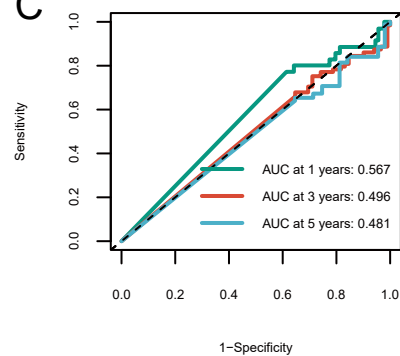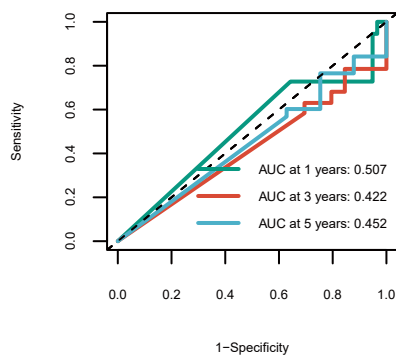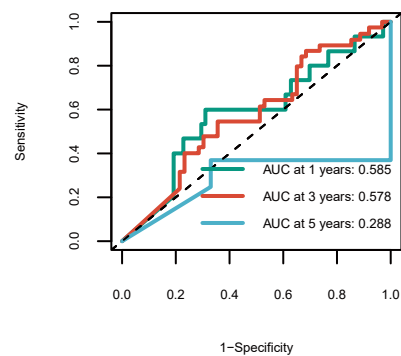**D**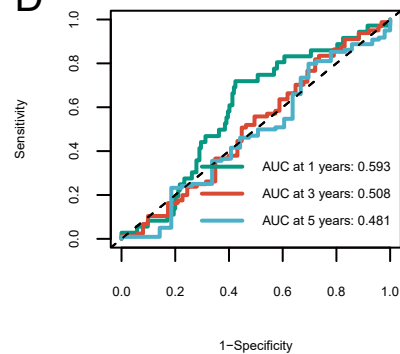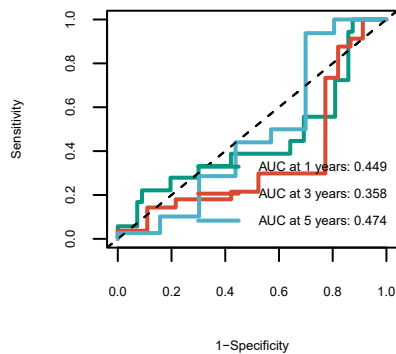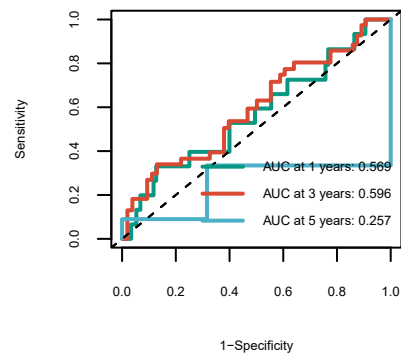

Supplement: Supplementary file 18 — Additional file 18: Figure S5. Internal and external dataset validation of ROC curves for immune cells at 1, 3, and 5 Years. (A) Immature B cell, (B) Activated CD4 T cell, (C) Central memory CD4 T cell, (D) Effector memory CD4 T cell. [file 12915_2024_1931_MOESM18_ESM.pdf]
