# Supplementary material for: A novel single-cell model reveals ferroptosis-associated biomarkers for individualized therapy and prognostic prediction in hepatocellular carcinoma
Source: BMC Biol. 2024 Jun 10;22:133. doi: 10.1186/s12915-024-01931-z (PMC11163722; doi:10.1186/s12915-024-01931-z)
Supplement: Supplementary file 20 — Additional file 20: Table S15. Table for the primer sequences used in qRT-PCR. [file 12915_2024_1931_MOESM20_ESM.docx]

| **Gene** | **Primer** | **Sequence** |
| --- | --- | --- |
| STMN1 | forward | 5′-GATTGTGCAGAATA- CACTGCCTGT-3′ |
|  | reverse | 5′-TTGCGTCTTTCTTCTGCAGCTTCT- 3′ |
| *S100A10* | forward | 5′-TCGCTGGGGATAAAGGCTAC-3′ |
|  | reverse | 5′-AAGAAGCTCTGGAAGCCCAC-3′ |
| FABP5 | forward | 5′-TGAAGGAGCTAGGAGTGGGAA-3′ |
|  | reverse | 5′-TGCACCATCTGTAAAGTTGCAG-3′ |
| CAPG | forward | 5’ AAGCTGAAGCC GGTGCCTGT -3′ |
|  | reverse | 5’ TGCTGGCCTATCCACAGGTGCA-3′ |
| *ANXA5* | forward | 5′-CATCTTTGGGACACGCAG-3′ |
|  | reverse | 5′-GGTCAAT CTCACTCCTC-3′ |
| UTRN | forward | 5′-CAAACACCCTCGACTTGGTT-3′ |
|  | reverse | 5′-TGGTGGAGCTGCTATCAGTG-3′ |
| ITM2A | forward | 5 ́-ATCCTGCAAATTCCCTTCGTG-3 ́ |
|  | reverse | 5 ́- CAGGTAAGCAGTCATTCCCTTT-3 ́ |
| ENO1 | forward | 5′-GCCGTGAACGAGAAGTCCTG-3′ |
|  | reverse | 5′-ACGCCTGAAGAGACTCGGT-3′ |
| CXCR3 | forward | 5′-GCTCTGAGGACTGCACCATTG-3′ |
|  | reverse | 5′-TGAAGTTTTAGTTTCCAAATGAGAAGGG-3′ |
| RGCC | forward | 5′-ACTCG GAGAG TGCAG ATT-3′ |
|  | reverse | 5′-TGACA GTGGC AGAGA GAA-3′ |
